# Supplementary material for: Association between COVID-19 outcomes and mask mandates, adherence, and attitudes
Source: PLoS One. 2021 Jun 23;16(6):e0252315. doi: 10.1371/journal.pone.0252315 (PMC8221503; doi:10.1371/journal.pone.0252315)
Supplement: S1 File — (PDF) [file pone.0252315.s001.pdf]

S1 File

A.1 Characteristics of COVID-19 outcome and adherence data

	Outcome			
	New confirmed COVID-19 cases	New confirmed deaths due to COVID-19	% of new hospital admissions with COVID-associated diagnoses	% of people who wore a mask for most or all of the time while in public in the past 5 days
**Source**	Delphi COVIDcast Epidata API	Delphi COVIDcast Epidata API	Delphi COVIDcast Epidata API	Delphi COVIDcast Epidata API
**Frequency**	Daily	Daily	Daily	Daily
**Geographical Resolution**	State-level	State-level	State-level	State-level
**Geographical Coverage**	All States and D.C.	All States and D.C.	All States and D.C.	Hawaii, Iowa, North Dakota, New Hampshire
**Date Range**	Feb. 1 - Sep 27 2020	Feb. 1 - Sep 27 2020	Feb. 1 - Sep 27 2020	Sep 8 - Nov 27, 2020
**Smoothing**	7-day average signal	7-day average signal	Systematic day-of-week effects removed	Seven day pooling
**Adjusted**	Per 100K population	Per 100K population	Per total admissions that day	Weighted to correct for a variety of biases
**Normalization**	Z-scored state-specific means and standard deviations	Z-scored state-specific means and standard deviations	Not Needed (already normalized as a percentage)	Z-scored state-specific means and standard deviations

S1 Table. Outcome data description. Characteristics of COVID-19 outcome variables

For the proportion of daily hospitalization admissions due to COVID-19, admissions are coded as COVID-19 related if the admission code U071, U072, B9729, or if primary ICD-10 code is R05, R060, R509, Z9911, R0902, R0603, R0609, R062, R069, R0602, R05, R0600, J9691, J9692, J9621, J9690, J9601, J9600, J189, J22, J1289, J129, J1281, B9721, B9732, B342, B349, A419, R531 or R6889 and there is a secondary ICD-10 code of Z20828, or if the primary ICD-10 code is Z20828.

A.2 Characteristics of COVID-19 control data

	Source	Freq.	Geograph. Resolution	Date Range	Normalization
**Retail+recreation mobility**	Google Mobility Reports	Daily	State-level	Feb 1-Sep 27 2020	Median day of the week baseline
**Grocery+pharmacy mobility**	Google Mobility Reports	Daily	State-level	Feb 1-Sep 27 2020	Median day of the week baseline
**Parks mobility**	Google Mobility Reports	Daily	State-level	Feb 1-Sep 27 2020	Median day of the week baseline
**Transit stations mobility**	Google Mobility Reports	Daily	State-level	Feb 1-Sep 27 2020	Median day of the week baseline
**Workplaces mobility**	Google Mobility Reports	Daily	State-level	Feb 1-Sep 27 2020	Median day of the week baseline
**Residential mobility**	Google Mobility Reports	Daily	State-level	Feb 1-Sep 27 2020	Median day of the week baseline
**Temperature**	NOAA	Daily	State-level	Feb 1-Sep 27 2020	Avg daily temperature
**Precipitation**	NOAA	Daily	State-level	Feb 1-Sep 27 2020	Avg daily precipitation
**Population Density**	Oxford World in Data	Daily	State-level	Feb 1-Sep 27 2020	Per square. km
**Human Develop. Index**	Oxford World in Data	Daily	Country-level	Feb 1-Sep 27 2020	Relative to UNDP limits
**New Test Rate**	COVID Tracking Project	Daily	Country-level	Feb 1-Sep 27 2020	Per 100K population
**Vaccination Rate**	Oxford World in Data	Daily	State-level	Jan 1-Mar 30 2021	Per 100 population

S2 Table. Control data description. Characteristics of control variables

A.3 State-level Mask Mandates Dates

State	Public Mask Mandates	Business Mask Mandates	State Ended Mask Mandates
Alabama	7/16/20	5/11/20	-
Alaska	4/24/20	4/24/20	5/22/20
Arizona	-	5/8/20	3/25/2021
Arkansas	7/20/20	5/11/20	3/30/2021
California	6/18/20	5/5/20	-
Colorado	7/16/20	4/23/20	-
Connecticut	4/20/20	4/3/20	-
Delaware	4/28/20	5/1/20	-
District of Columbia	4/17/20	4/15/20	-
Florida	-	5/11/20	-
Georgia	-	4/27/20	-
Hawaii	11/16/20	4/16/20	-
Idaho	-	-	-
Illinois	5/1/20	5/1/20	-
Indiana	7/27/20	5/1/20	-
Iowa	-	11/16/20	2/7/2021
Kansas	7/3/20	7/3/20	-
Kentucky	5/11/20	5/11/20	-
Louisiana	-	5/1/20	-
Maine	5/1/20	5/1/20	-
Maryland	4/18/20	4/18/20	-
Massachusetts	5/6/20	5/6/20	-
Michigan	4/27/20	4/26/20	10/2/2020
Minnesota	7/24/20	6/1/20	-
Mississippi	8/5/20	5/7/20	9/30/20
Missouri	-	-	-
Montana	-	-	2/12/2021
Nebraska	-	5/4/20	-
Nevada	6/26/20	5/9/20	-
New Hampshire	11/20/20	5/1/20	-
New Jersey	4/8/20	4/8/20	-
New Mexico	5/15/20	5/6/20	-
New York	4/17/20	4/17/20	-
North Carolina	6/26/20	6/26/20	-
North Dakota	11/14/20	4/28/20	1/18/2021
Ohio	7/23/20	4/29/20	-
Oklahoma	-	-	-
Oregon	7/1/20	5/9/20	-
Pennsylvania	7/1/20	4/19/20	-
Rhode Island	4/18/20	4/18/20	-
South Carolina	-	8/3/20	-
South Dakota	-	-	-
Tennessee	-	-	-
Texas	7/3/20	5/8/20	3/10/2021
Utah	4/10/20	4/10/20	5/1/20
Vermont	8/1/20	4/17/20	-
Virginia	5/29/20	5/29/20	-
Washington	6/26/20	5/4/20	-
West Virginia	7/7/20	5/4/20	-
Wisconsin	8/1/20	8/1/20	3/31/2021
Wyoming	-	5/1/20	3/16/2021

S3 Table. Mandate data description. Mask mandate start and end dates as of March 30, 2021

A.4 Comparing controlling for outcome delay and growth

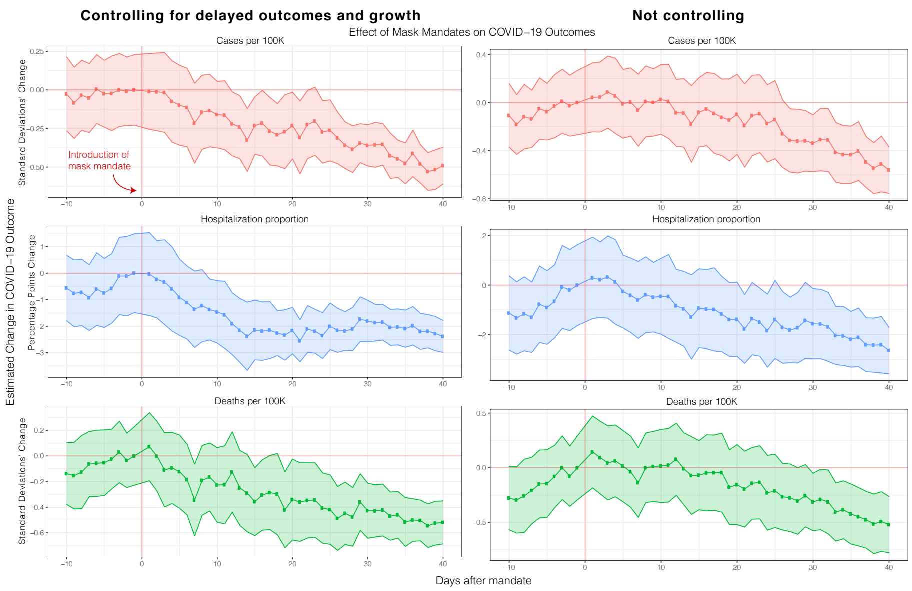

S1 Fig. Delay robustness check. Robustness check of main event study results (section fig. 1) where we compare controlling for past outcome values (with a delay of 14 days) and growth rates as a way to minimize confounding due to peoples private behavioral changes to COVID-19 [35]. As can be see in the figure, the overall trajectory and magnitude of the treatment effect is consistent with our previous main result. Full regression results are shown in S8 Table.

A.5 Testing rate

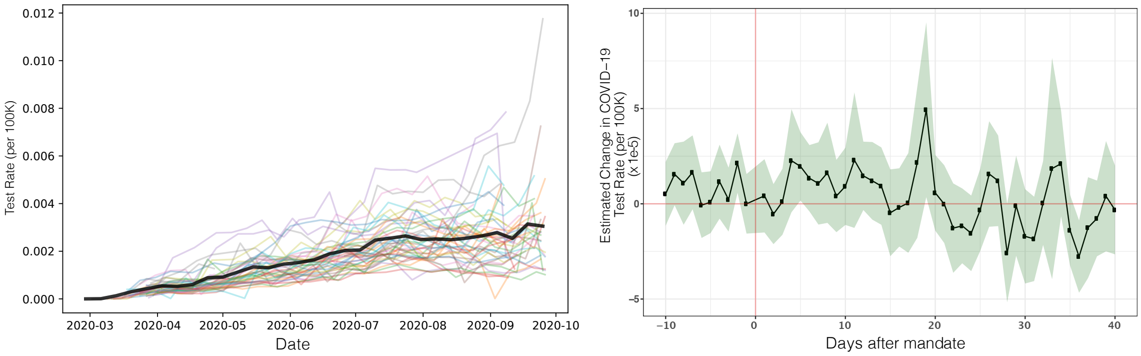

S2 Fig. Testing rate robustness check. Left: Per state (each state a different color) and average (over all states, thicker black line) testing rate increased during our period of investigation. **Right:** Event study of testing rate over all states controlling for a number of factors showing that mask mandates are not associated with changes in testing rate during our period of investigation. This suggests that testing rate decreases are not behind the decrease in COVID-19 outcomes we observe after the introduction of mask mandates. Full regression results are shown in S13 Table.

A.6 County-level heterogeneity

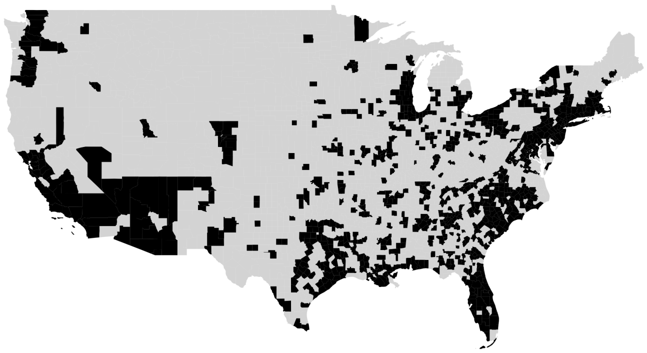

S3 Fig. County map. Map showing the 857 counties for which we have outcome and control variable data. Missing counties either had too few cases (and therefore cannot be reported for privacy reasons) or had data available later than the end of a mask mandate ended. The counties we have data for contain 77% of the US population (as per the 2018 US Census).

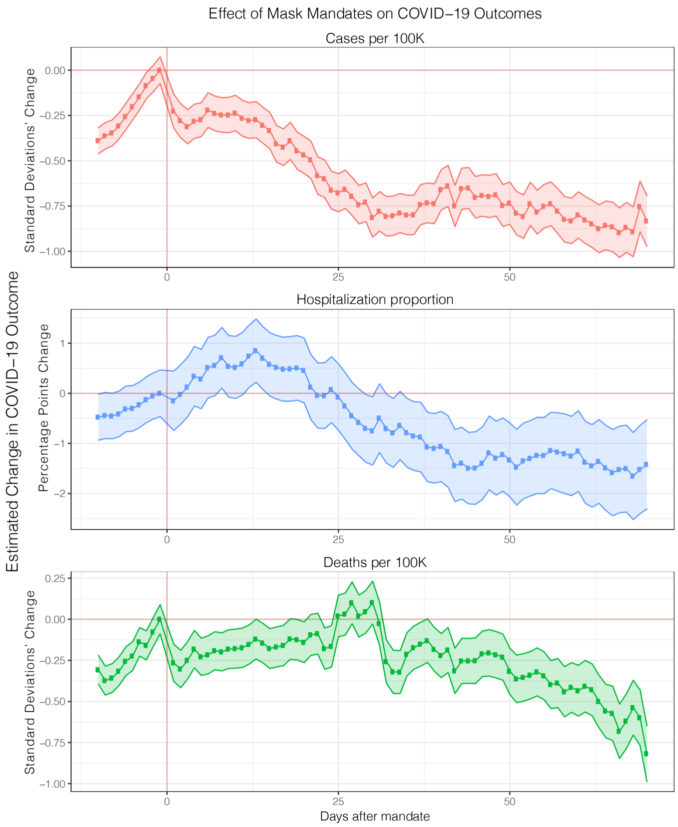

S4 Fig. County-level robustness check. Robustness check of main event study results (section Fig 1) where we focus on county level mandates only. Full regression results are shown in S11 Table.

A.7 Pre-trends

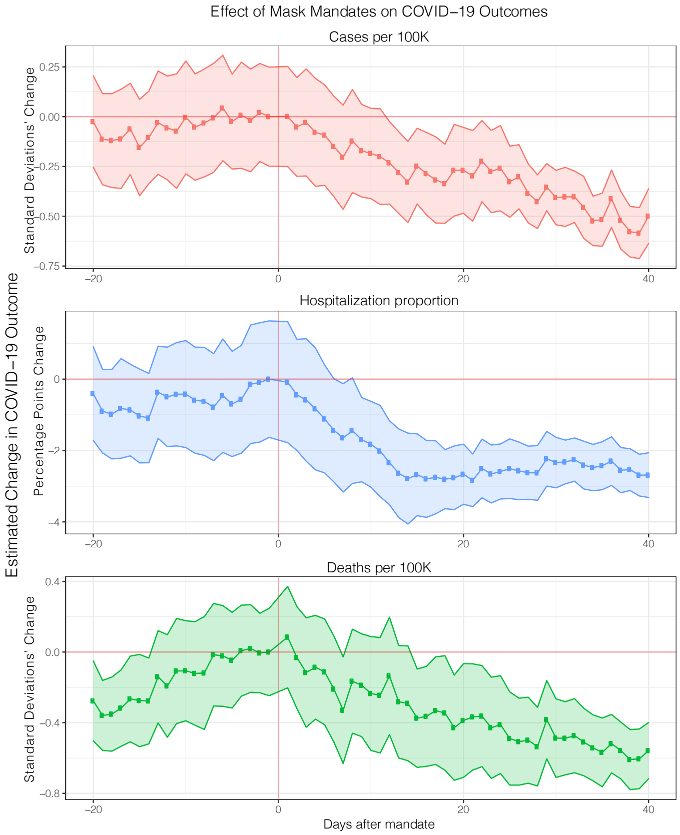

S5 Fig. Pre-trends robustness check. Robustness check of main event study results (section Fig 1) where we investigate longer pre-treatment windows and no fixed effect.

A.8 Controlling for confirmed cases

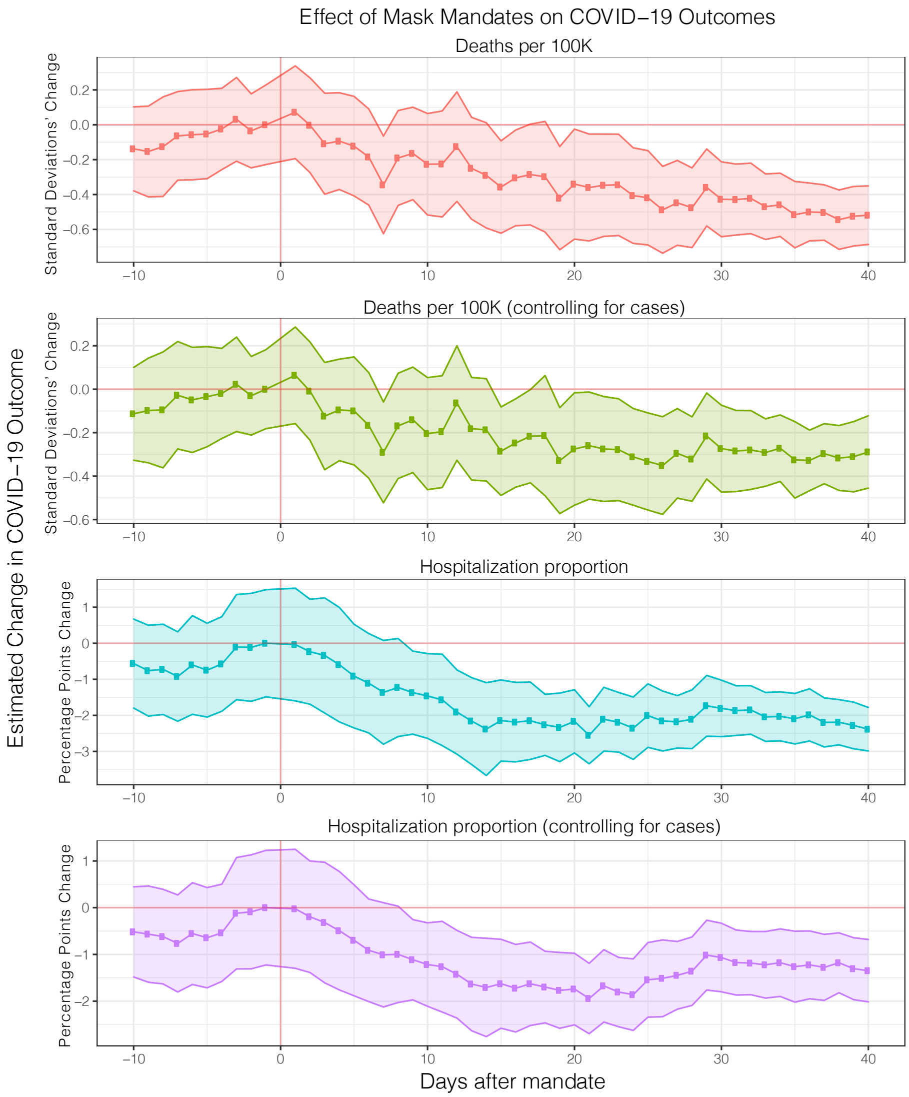

S6 Fig. Controlling cases robustness check. Robustness check of main event study results (section Fig 1) where we compare the effect of mask mandates on deaths and hospitalization with and without controlling for cases. Because the trajectory and magnitude of the treatment effect is consistent with our previous main result under either specification (with and without controlling for cases), this demonstrates that our main result is not sensitive to whether we account for the cases underlying the more severe outcomes. Full regression results are shown in S7 Table.

A.9 Mask adherence following mask mandate introduction

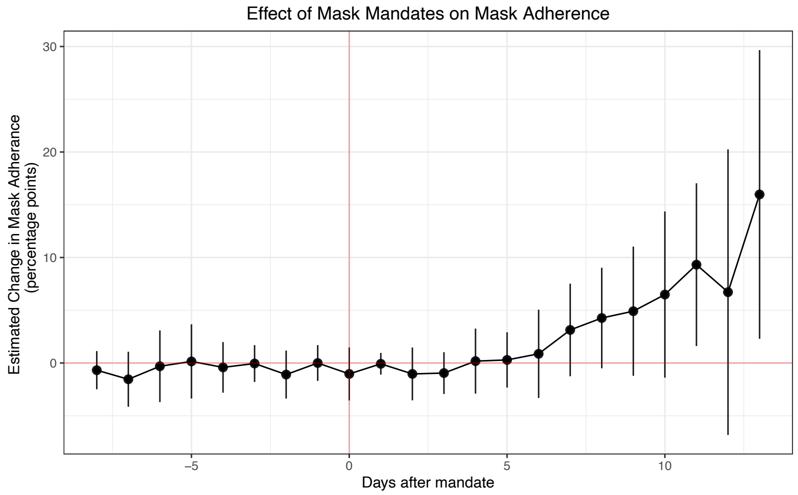

S7 Fig. Adherence introducing mandates robustness check. Event study of mask mandate introduction when not controlling for cases, deaths and testing rate. As expected, the treatment effect becomes noisier but reamins consistent to when controlling for cases, deaths and testing rate. Full regression results are shown in S14 Table.

A.10 Mask adherence following mask mandate lifting

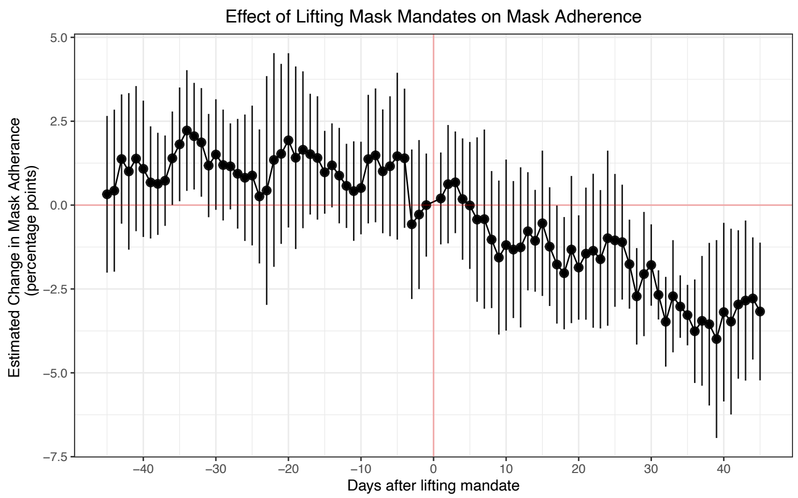

S8 Fig. Adherence lifting mandates robustness check. Event study of mask mandate lifting when not controlling for cases, deaths and testing rate. Full regression results are shown in S15 Table.

A.11 International Community Mask Adherence and Attitudes

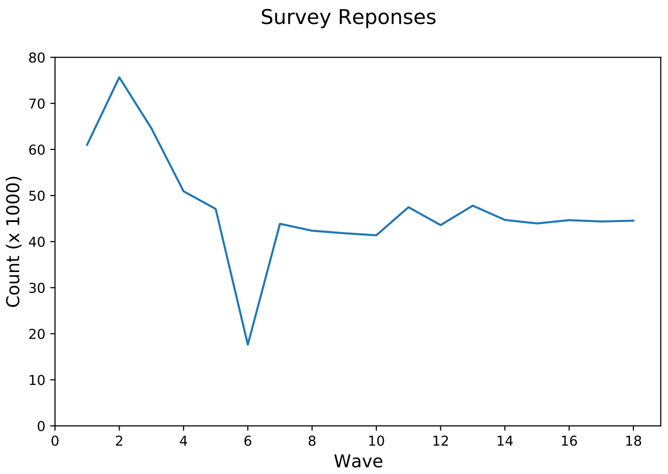

S9 Fig. Survey response counts. Number of responses per wave of survey.

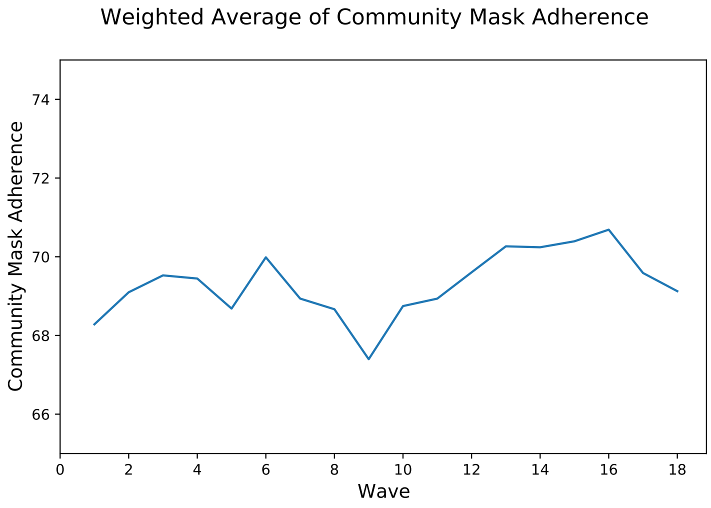

S10 Fig. Survey average adherence. Average (weighted) community mask adherence responseper wave.

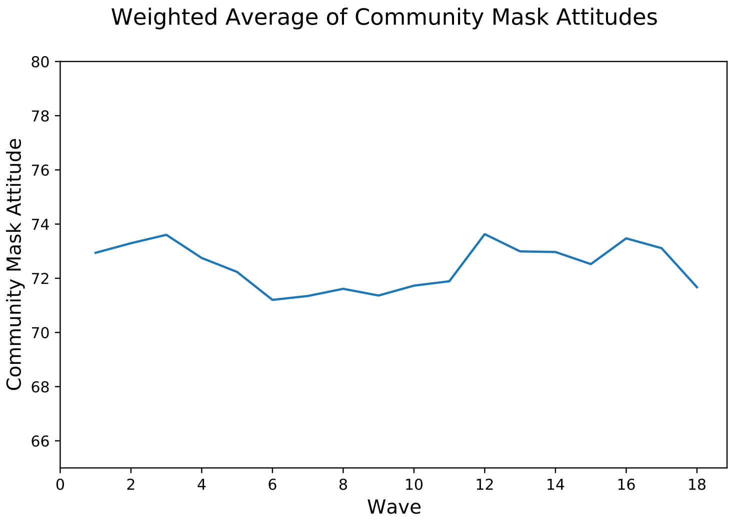

S11 Fig. Survey average attitudes. Average (weighted) community mask attitude response perwave.

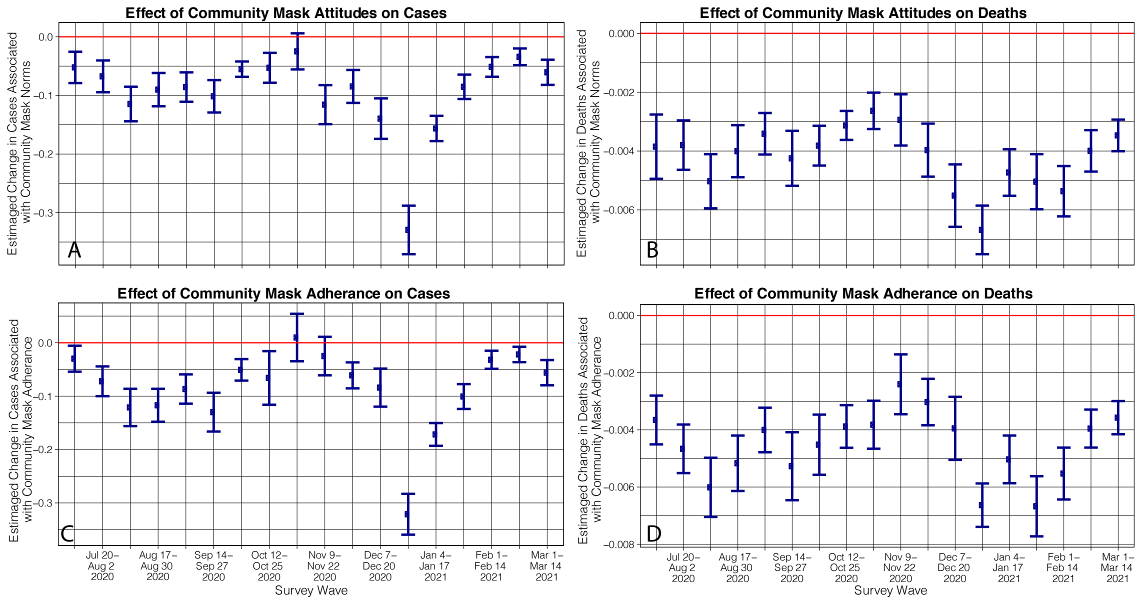

S12 Fig. Individual waves robustness check. Dis-aggregated (by survey wave) estimates of the effect of community mask attitudes on COVID-19 cases **(A)** and deaths **(B)**, and community mask adherence on COVID-19 cases **(C)** and deaths **(D)** in a 68-country survey with 479K responses. Error bars show a 95% confidence interval. Full regression statistics are shown in S18, S19, S20 and S21 Tables.

A.12 Event study and multi-linear regression tables

S4 Table. Regression table. Event Study regression summary statistics for main result (section fig. 1)

	Dependent variable:		
	confirmed cases	deaths	hospitalization
	(1)	(2)	(3)
confirmed cases delay	0.483***		
	(0.025)		
confirmed cases growth	−0.00000		
	(0.00002)		
deaths delay		0.507***	
		(0.031)	
deaths growth		0.001***	
		(0.0002)	
hospitalization delay			0.549***
			(0.027)
hospitalization growth			0.109***
			(0.032)
new test rate	56.176***	27.940**	154.787***
	(14.681)	(10.586)	(43.311)
precipitation	−0.001	−0.004***	0.002
	(0.001)	(0.001)	(0.006)
temperature	0.016***	0.002	0.055***
	(0.003)	(0.004)	(0.015)
retail and recreation	−7.824***	−2.338	−35.993***
	(1.796)	(1.539)	(11.299)
grocery and pharmacy	5.833**	−0.025	12.745
	(2.306)	(2.015)	(14.192)
parks	0.388***	0.259***	1.210***
	(0.096)	(0.074)	(0.319)
transit stations	−1.211**	0.007	−4.089*

	(0.509)	(0.423)	(2.081)
workplaces	1.490*	2.085***	10.502***
	(0.816)	(0.704)	(3.450)
residential	−43.630***	−29.042***	−279.358***
	(7.292)	(7.774)	(52.035)
grocery and pharmacy squared	−2.864***	−0.308	−5.955
	(1.065)	(0.916)	(6.241)
retail and recreation squared	3.560***	0.390	16.584***
	(1.011)	(0.860)	(5.968)
parks squared	−0.086***	−0.041**	−0.233***
	(0.019)	(0.016)	(0.064)
transit stations squared	0.959***	0.168	2.857**
	(0.342)	(0.241)	(1.187)
workplaces squared	−1.461**	−1.463***	−9.818***
	(0.553)	(0.512)	(2.276)
residential squared	20.728***	14.635***	129.688***
	(3.180)	(3.471)	(23.620)
stay-at-home day	0.0001**	0.0001**	−0.00000
	(0.00003)	(0.00003)	(0.0001)
business closure day	0.0001*	0.0001***	−0.00001
	(0.00003)	(0.00003)	(0.0001)
day -10	0.373***	0.478***	1.732***
	(0.122)	(0.123)	(0.629)
day -9	0.316**	0.463***	1.534**
	(0.118)	(0.133)	(0.642)
day -8	0.365***	0.491***	1.571**
	(0.116)	(0.146)	(0.639)
day -7	0.347***	0.553***	1.372**
	(0.114)	(0.130)	(0.632)
day -6	0.405***	0.559***	1.693**
	(0.114)	(0.132)	(0.698)

day -5	0.375***	0.564***	1.549**
	(0.110)	(0.131)	(0.664)
day -4	0.377***	0.592***	1.720**
	(0.123)	(0.119)	(0.669)
day -3	0.399***	0.648***	2.189***
	(0.121)	(0.123)	(0.744)
day -2	0.391***	0.582***	2.181***
	(0.113)	(0.108)	(0.764)
day -1	0.400***	0.617***	2.294***
	(0.117)	(0.116)	(0.758)
day 1	0.389***	0.688***	2.260***
	(0.125)	(0.136)	(0.797)
day 2	0.387***	0.614***	2.060***
	(0.129)	(0.139)	(0.743)
day 3	0.382***	0.508***	1.956**
	(0.132)	(0.148)	(0.815)
day 4	0.325**	0.523***	1.701**
	(0.135)	(0.142)	(0.810)
day 5	0.300**	0.494***	1.384*
	(0.132)	(0.146)	(0.733)
day 6	0.285**	0.432***	1.186*
	(0.130)	(0.141)	(0.703)
day 7	0.184	0.272*	0.934
	(0.132)	(0.143)	(0.734)
day 8	0.255**	0.426***	1.068
	(0.122)	(0.139)	(0.693)
day 9	0.265**	0.452***	0.925
	(0.120)	(0.135)	(0.587)
day 10	0.239**	0.390**	0.833
	(0.110)	(0.149)	(0.600)

day 11	0.236**	0.392**	0.724
	(0.114)	(0.155)	(0.646)
day 12	0.180*	0.491***	0.388
	(0.106)	(0.160)	(0.596)
day 13	0.158	0.367**	0.137
	(0.104)	(0.149)	(0.615)
day 14	0.075	0.326**	−0.085
	(0.104)	(0.154)	(0.657)
day 15	0.168*	0.259*	0.152
	(0.095)	(0.135)	(0.574)
day 16	0.184*	0.312**	0.108
	(0.109)	(0.140)	(0.562)
day 17	0.132	0.331**	0.146
	(0.112)	(0.148)	(0.547)
day 18	0.109	0.318*	0.033
	(0.103)	(0.162)	(0.432)
day 19	0.129	0.196	−0.038
	(0.122)	(0.151)	(0.485)
day 20	0.168	0.276*	0.130
	(0.110)	(0.161)	(0.447)
day 21	0.091	0.257	−0.257
	(0.121)	(0.156)	(0.404)
day 22	0.175	0.270*	0.188
	(0.113)	(0.150)	(0.451)
day 23	0.197*	0.272*	0.104
	(0.113)	(0.148)	(0.421)
day 24	0.127	0.210	−0.060
	(0.103)	(0.140)	(0.441)
day 25	0.137	0.198	0.289
	(0.101)	(0.138)	(0.449)
day 26	0.088	0.129	0.139

	(0.085)	(0.127)	(0.424)
day 27	0.041	0.169	0.116
	(0.077)	(0.124)	(0.371)
day 28	0.015	0.141	0.188
	(0.077)	(0.117)	(0.415)
day 29	0.054	0.257**	0.560
	(0.064)	(0.113)	(0.430)
day 30	0.040	0.189*	0.489
	(0.068)	(0.110)	(0.400)
day 31	0.044	0.187*	0.428
	(0.074)	(0.104)	(0.352)
day 32	0.046	0.194*	0.445
	(0.069)	(0.103)	(0.344)
day 33	−0.027	0.147	0.252
	(0.075)	(0.096)	(0.346)
day 34	−0.047	0.157*	0.268
	(0.062)	(0.093)	(0.347)
day 35	−0.076	0.101	0.202
	(0.068)	(0.097)	(0.357)
day 36	−0.012	0.116	0.309
	(0.077)	(0.085)	(0.369)
day 37	−0.080	0.113	0.100
	(0.064)	(0.081)	(0.347)
day 38	−0.130**	0.072	0.105
	(0.062)	(0.087)	(0.320)
day 39	−0.117*	0.092	0.016
	(0.066)	(0.087)	(0.330)
day 40	−0.092	0.098	−0.089
	(0.061)	(0.086)	(0.307)
Constant	21.774***	13.914***	157.262***
	(4.579)	(4.614)	(31.907)

Observations	10,078	10,078	10,078
R^2	0.636	0.543	0.702
Adjusted R^2	0.632	0.537	0.698
Residual Std. Error (df = 9956)	0.606	0.678	2.675

Note: *p<0.1; **p<0.05; ***p<0.01

S5 Table. Regression table. Event Study regression summary statistics for robustness check (fig. 2) where we only consider mask mandates that require the public to wear masks.

	Dependent variable:		
	confirmed cases	deaths	hospitalization
	(1)	(2)	(3)
confirmed cases delay	0.456^{***}		
	(0.026)		
confirmed cases growth	-0.00000		
	(0.00002)		
deaths delay		0.535^{***}	
		(0.032)	
deaths growth		0.001^{***}	
		(0.0002)	
hospitalization delay			0.536^{***}
			(0.027)
hospitalization growth			0.095^{***}
			(0.031)
new test rate	56.134^{***}	29.252^{***}	153.519^{***}
	(14.218)	(10.493)	(41.989)
precipitation	-0.001	-0.004^{***}	0.001
	(0.001)	(0.001)	(0.006)
temperature	0.013^{***}	0.001	0.048^{***}
	(0.003)	(0.004)	(0.015)
retail and recreation	-6.802^{***}	-1.546	-32.481^{***}
	(1.683)	(1.585)	(10.657)
grocery and pharmacy	4.819^{**}	-0.665	10.359
	(2.277)	(1.984)	(13.586)
parks	0.349^{***}	0.255^{***}	1.097^{***}
	(0.093)	(0.074)	(0.310)
transit stations	-0.837^{*}	0.114	-3.267
	(0.473)	(0.415)	(2.046)

workplaces	1.123	2.323***	10.197***
	(0.773)	(0.722)	(3.331)
residential	−38.867***	−28.011***	−263.254***
	(7.584)	(8.089)	(47.976)
grocery and pharmacy squared	−2.421**	−0.016	−4.887
	(1.039)	(0.898)	(5.976)
retail and recreation squared	3.031***	−0.075	14.553**
	(0.946)	(0.885)	(5.616)
parks squared	−0.076***	−0.038**	−0.199***
	(0.019)	(0.017)	(0.063)
transit stations squared	0.643**	0.039	2.056*
	(0.306)	(0.237)	(1.145)
workplaces squared	−1.122**	−1.599***	−9.348***
	(0.533)	(0.533)	(2.169)
residential squared	18.581***	14.312***	122.574***
	(3.308)	(3.598)	(21.793)
stay-at-home day	0.0001**	0.0001**	−0.00002
	(0.00003)	(0.00003)	(0.0001)
business closure day	0.0001**	0.0001***	0.0001
	(0.00003)	(0.00003)	(0.0001)
day -10	0.530***	0.290**	2.672***
	(0.115)	(0.139)	(0.791)
day -9	0.537***	0.265*	2.820***
	(0.117)	(0.137)	(0.878)
day -8	0.541***	0.243*	2.624***
	(0.109)	(0.142)	(0.797)
day -7	0.572***	0.314**	2.536***
	(0.112)	(0.131)	(0.773)
day -6	0.615***	0.377***	2.736***
	(0.135)	(0.137)	(0.930)
day -5	0.649***	0.451***	2.802***

	(0.131)	(0.129)	(0.877)
day -4	0.602***	0.494***	2.715***
	(0.133)	(0.125)	(0.834)
day -3	0.625***	0.477***	3.082***
	(0.124)	(0.136)	(0.901)
day -2	0.645***	0.564***	3.125***
	(0.127)	(0.125)	(0.863)
day -1	0.679***	0.542***	3.029***
	(0.128)	(0.124)	(0.828)
day 1	0.668***	0.494***	2.610***
	(0.127)	(0.145)	(0.768)
day 2	0.704***	0.398***	2.510***
	(0.137)	(0.140)	(0.730)
day 3	0.653***	0.236	2.237***
	(0.133)	(0.157)	(0.778)
day 4	0.663***	0.330**	2.085***
	(0.132)	(0.143)	(0.709)
day 5	0.647***	0.357**	2.018***
	(0.131)	(0.147)	(0.673)
day 6	0.615***	0.341**	1.741**
	(0.131)	(0.148)	(0.673)
day 7	0.561***	0.296*	1.661**
	(0.133)	(0.154)	(0.725)
day 8	0.576***	0.356**	1.642**
	(0.137)	(0.155)	(0.716)
day 9	0.600***	0.403***	1.718***
	(0.134)	(0.137)	(0.595)
day 10	0.537***	0.334**	1.439**
	(0.130)	(0.149)	(0.633)
day 11	0.544***	0.295*	1.371**
	(0.129)	(0.157)	(0.594)

day 12	0.507***	0.314*	1.093*
	(0.121)	(0.160)	(0.551)
day 13	0.488***	0.205	0.768
	(0.122)	(0.148)	(0.581)
day 14	0.438***	0.207	0.692
	(0.134)	(0.155)	(0.580)
day 15	0.488***	0.234	1.059*
	(0.129)	(0.143)	(0.528)
day 16	0.469***	0.260**	0.913*
	(0.140)	(0.127)	(0.480)
day 17	0.486***	0.235*	0.901*
	(0.140)	(0.130)	(0.512)
day 18	0.412***	0.235	0.610
	(0.140)	(0.156)	(0.454)
day 19	0.437***	0.121	0.371
	(0.144)	(0.132)	(0.524)
day 20	0.430***	0.117	0.399
	(0.140)	(0.121)	(0.459)
day 21	0.385**	0.100	0.155
	(0.148)	(0.124)	(0.473)
day 22	0.480***	0.130	0.472
	(0.157)	(0.120)	(0.452)
day 23	0.501***	0.187*	0.512
	(0.166)	(0.111)	(0.444)
day 24	0.393***	0.141	0.341
	(0.146)	(0.109)	(0.427)
day 25	0.357**	0.184	0.518
	(0.142)	(0.118)	(0.439)
day 26	0.348**	0.195	0.574
	(0.139)	(0.128)	(0.425)

day 27	0.203	0.222*	0.462
	(0.136)	(0.132)	(0.403)
day 28	0.149	0.183	0.453
	(0.127)	(0.128)	(0.476)
day 29	0.190	0.326**	0.813*
	(0.121)	(0.138)	(0.436)
day 30	0.154	0.266*	0.591
	(0.124)	(0.132)	(0.409)
day 31	0.092	0.227**	0.383
	(0.125)	(0.111)	(0.402)
day 32	0.126	0.270**	0.399
	(0.116)	(0.115)	(0.400)
day 33	0.031	0.170	0.154
	(0.121)	(0.131)	(0.433)
day 34	0.042	0.144	0.171
	(0.111)	(0.134)	(0.419)
day 35	−0.052	0.107	−0.119
	(0.112)	(0.126)	(0.444)
day 36	−0.010	0.176	0.024
	(0.120)	(0.117)	(0.452)
day 37	−0.022	0.219*	−0.135
	(0.112)	(0.124)	(0.427)
day 38	−0.050	0.108	−0.270
	(0.104)	(0.105)	(0.410)
day 39	−0.089	0.056	−0.473
	(0.100)	(0.107)	(0.443)
day 40	−0.062	0.001	−0.615
	(0.084)	(0.100)	(0.370)
Constant	19.299***	13.273***	148.346***
	(4.764)	(4.744)	(29.415)
Observations	10,078	10,078	10,078

R^2	0.654	0.533	0.708
Adjusted R^2	0.649	0.527	0.705
Residual Std. Error (df = 9956)	0.591	0.686	2.646
Note:			*p<0.1; **p<0.05; ***p<0.01

S6 Table. Regression table. Event Study regression summary statistics for robustness check (fig. 2) where we only consider mask mandates that require business employees to wear masks.

	Dependent variable:		
	confirmed cases	deaths	hospitalization
	(1)	(2)	(3)
confirmed cases delay	0.484***		
	(0.025)		
confirmed cases growth	−0.00000		
	(0.00002)		
deaths delay		0.507***	
		(0.031)	
deaths growth		0.001***	
		(0.0002)	
hospitalization delay			0.549***
			(0.027)
hospitalization growth			0.108***
			(0.032)
new test rate	56.130***	27.994**	154.780***
	(14.672)	(10.586)	(43.300)
precipitation	−0.001	−0.004***	0.002
	(0.001)	(0.001)	(0.006)
temperature	0.016***	0.002	0.055***
	(0.003)	(0.004)	(0.015)
retail and recreation	−7.807***	−2.328	−35.948***
	(1.799)	(1.541)	(11.343)
grocery and pharmacy	5.806**	−0.059	12.629
	(2.311)	(2.016)	(14.240)
parks	0.389***	0.258***	1.217***
	(0.096)	(0.074)	(0.319)
transit stations	−1.213**	0.007	−4.111*
	(0.509)	(0.423)	(2.082)

workplaces	1.498*	2.093***	10.571***
	(0.817)	(0.704)	(3.451)
residential	−43.651***	−29.103***	−280.024***
	(7.292)	(7.766)	(52.177)
grocery and pharmacy squared	−2.851**	−0.293	−5.894
	(1.067)	(0.916)	(6.264)
retail and recreation squared	3.552***	0.385	16.549***
	(1.013)	(0.861)	(5.995)
parks squared	−0.086***	−0.041**	−0.235***
	(0.019)	(0.016)	(0.064)
transit stations squared	0.959***	0.167	2.864**
	(0.342)	(0.241)	(1.187)
workplaces squared	−1.461**	−1.465***	−9.854***
	(0.554)	(0.512)	(2.282)
residential squared	20.749***	14.671***	130.035***
	(3.182)	(3.468)	(23.686)
stay-at-home day	0.0001**	0.0001**	−0.00000
	(0.00003)	(0.00003)	(0.0001)
business closure day	0.0001*	0.0001***	−0.00001
	(0.00003)	(0.00003)	(0.0001)
day -10	0.359***	0.483***	1.785***
	(0.121)	(0.124)	(0.655)
day -9	0.319***	0.472***	1.573**
	(0.119)	(0.134)	(0.662)
day -8	0.367***	0.494***	1.579**
	(0.116)	(0.146)	(0.642)
day -7	0.337***	0.541***	1.296**
	(0.114)	(0.129)	(0.597)
day -6	0.392***	0.558***	1.653**
	(0.112)	(0.132)	(0.679)
day -5	0.388***	0.580***	1.478**

	(0.113)	(0.134)	(0.632)
day -4	0.399***	0.609***	1.689**
	(0.127)	(0.121)	(0.657)
day -3	0.436***	0.657***	2.106***
	(0.130)	(0.124)	(0.711)
day -2	0.390***	0.579***	2.076***
	(0.113)	(0.108)	(0.734)
day -1	0.397***	0.617***	2.140***
	(0.116)	(0.117)	(0.721)
day 1	0.386***	0.690***	2.156***
	(0.125)	(0.136)	(0.779)
day 2	0.375***	0.619***	1.973***
	(0.126)	(0.140)	(0.736)
day 3	0.364***	0.509***	1.870**
	(0.128)	(0.148)	(0.811)
day 4	0.284**	0.516***	1.573*
	(0.129)	(0.140)	(0.803)
day 5	0.284**	0.474***	1.255*
	(0.130)	(0.142)	(0.737)
day 6	0.281**	0.420***	1.084
	(0.129)	(0.138)	(0.706)
day 7	0.196	0.268*	0.821
	(0.133)	(0.142)	(0.742)
day 8	0.273**	0.439***	1.047
	(0.126)	(0.141)	(0.697)
day 9	0.254**	0.444***	0.906
	(0.119)	(0.134)	(0.589)
day 10	0.241**	0.375**	0.829
	(0.110)	(0.147)	(0.601)
day 11	0.221*	0.378**	0.700
	(0.111)	(0.153)	(0.649)

day 12	0.180*	0.494***	0.403
	(0.106)	(0.161)	(0.596)
day 13	0.158	0.382**	0.216
	(0.104)	(0.151)	(0.614)
day 14	0.067	0.329**	−0.022
	(0.103)	(0.155)	(0.657)
day 15	0.170*	0.272*	0.187
	(0.095)	(0.138)	(0.578)
day 16	0.194*	0.326**	0.095
	(0.111)	(0.143)	(0.562)
day 17	0.147	0.350**	0.119
	(0.115)	(0.151)	(0.545)
day 18	0.132	0.324*	−0.010
	(0.109)	(0.163)	(0.427)
day 19	0.120	0.192	−0.090
	(0.120)	(0.150)	(0.480)
day 20	0.157	0.266	0.129
	(0.108)	(0.159)	(0.447)
day 21	0.061	0.231	−0.249
	(0.113)	(0.152)	(0.405)
day 22	0.155	0.243	0.278
	(0.109)	(0.146)	(0.470)
day 23	0.197*	0.256*	0.168
	(0.113)	(0.146)	(0.438)
day 24	0.115	0.198	−0.045
	(0.103)	(0.139)	(0.445)
day 25	0.132	0.191	0.283
	(0.100)	(0.138)	(0.447)
day 26	0.066	0.117	0.088
	(0.081)	(0.126)	(0.409)

day 27	0.027	0.159	0.070
	(0.077)	(0.124)	(0.360)
day 28	−0.009	0.136	0.061
	(0.078)	(0.117)	(0.388)
day 29	0.049	0.258**	0.487
	(0.064)	(0.113)	(0.420)
day 30	0.032	0.201*	0.455
	(0.069)	(0.110)	(0.396)
day 31	0.046	0.192*	0.429
	(0.074)	(0.104)	(0.353)
day 32	0.033	0.198*	0.427
	(0.070)	(0.103)	(0.339)
day 33	−0.027	0.141	0.215
	(0.075)	(0.095)	(0.339)
day 34	−0.053	0.171*	0.190
	(0.063)	(0.094)	(0.340)
day 35	−0.082	0.104	0.098
	(0.068)	(0.097)	(0.353)
day 36	−0.016	0.137	0.217
	(0.078)	(0.087)	(0.370)
day 37	−0.083	0.122	0.032
	(0.064)	(0.083)	(0.356)
day 38	−0.138**	0.081	0.046
	(0.064)	(0.088)	(0.331)
day 39	−0.127*	0.102	−0.044
	(0.068)	(0.090)	(0.340)
day 40	−0.098	0.099	−0.155
	(0.062)	(0.086)	(0.323)
Constant	21.776***	13.946***	157.608***
	(4.576)	(4.609)	(31.988)
Observations	10,078	10,078	10,078

R^2	0.636	0.543	0.701
Adjusted R^2	0.632	0.537	0.698
Residual Std. Error (df = 9956)	0.606	0.678	2.678
Note:			*p<0.1; **p<0.05; ***p<0.01

S7 Table. Regression table. Event Study regression summary statistics for robustness check (S6. Fig) where we compare the effect of mask mandates on deaths and hospitalization with and without controlling for cases.

	Dependent variable:			
	deaths	deaths	hosp.	hosp.
	(1)	(2)	(3)	(4)
confirmed cases		0.410***		1.990***
		(0.022)		(0.243)
deaths delay	0.507***	0.439***		
	(0.031)	(0.031)		
deaths growth	0.001***	0.001***		
	(0.0002)	(0.0001)		
hospitalization delay			0.549***	0.387***
			(0.027)	(0.027)
hospitalization growth			0.109***	0.082***
			(0.032)	(0.030)
new test rate	27.940**	−1.096	154.787***	31.262
	(10.586)	(6.646)	(43.311)	(38.780)
precipitation	−0.004***	−0.004***	0.002	0.005
	(0.001)	(0.001)	(0.006)	(0.006)
temperature	0.002	−0.003	0.055***	0.023
	(0.004)	(0.004)	(0.015)	(0.015)
retail & recreation	−2.338	1.218	−35.993***	−20.360**
	(1.539)	(1.097)	(11.299)	(9.758)
grocery & pharmacy	−0.025	−2.114	12.745	5.766
	(2.015)	(1.760)	(14.192)	(12.601)
parks	0.259***	0.056	1.210***	0.339
	(0.074)	(0.069)	(0.319)	(0.316)
transit stations	0.007	0.413	−4.089*	−2.765
	(0.423)	(0.441)	(2.081)	(1.875)
workplaces	2.085***	2.317***	10.502***	12.020***
	(0.704)	(0.549)	(3.450)	(2.733)

residential	-29.042^{***}	-2.983	-279.358^{***}	-174.851^{***}
	(7.774)	(7.367)	(52.035)	(42.791)
grocery & pharmacy squared	-0.308	0.733	-5.955	-2.425
	(0.916)	(0.822)	(6.241)	(5.565)
retail & recreation squared	0.390	-1.315^{**}	16.584^{***}	8.999^{*}
	(0.860)	(0.640)	(5.968)	(5.230)
parks squared	-0.041^{**}	0.011	-0.233^{***}	-0.001
	(0.016)	(0.017)	(0.064)	(0.067)
transit stations squared	0.168	-0.181	2.857^{**}	1.532
	(0.241)	(0.261)	(1.187)	(1.173)
workplaces squared	-1.463^{***}	-1.471^{***}	-9.818^{***}	-10.316^{***}
	(0.512)	(0.404)	(2.276)	(1.907)
residential squared	14.635^{***}	2.518	129.688^{***}	80.931^{***}
	(3.471)	(3.255)	(23.620)	(19.252)
stay-at-home day	0.0001^{**}	0.00003	-0.00000	-0.0002
	(0.00003)	(0.00003)	(0.0001)	(0.0001)
business closure day	0.0001^{***}	0.0001^{***}	-0.00001	-0.0001
	(0.00003)	(0.00003)	(0.0001)	(0.0001)
day -10	0.478^{***}	0.310^{***}	1.732^{***}	1.042^{**}
	(0.123)	(0.109)	(0.629)	(0.492)
day -9	0.463^{***}	0.325^{**}	1.534^{**}	0.995^{*}
	(0.133)	(0.123)	(0.642)	(0.525)
day -8	0.491^{***}	0.328^{**}	1.571^{**}	0.942^{*}
	(0.146)	(0.136)	(0.639)	(0.516)
day -7	0.553^{***}	0.395^{***}	1.372^{**}	0.795
	(0.130)	(0.126)	(0.632)	(0.530)
day -6	0.559^{***}	0.374^{***}	1.693^{**}	1.007^{*}
	(0.132)	(0.123)	(0.698)	(0.556)
day -5	0.564^{***}	0.389^{***}	1.549^{**}	0.918^{*}
	(0.131)	(0.118)	(0.664)	(0.547)

day -4	0.592***	0.404***	1.720**	1.022*
	(0.119)	(0.106)	(0.669)	(0.531)
day -3	0.648***	0.446***	2.189***	1.442**
	(0.123)	(0.111)	(0.744)	(0.608)
day -2	0.582***	0.393***	2.181***	1.472**
	(0.108)	(0.092)	(0.764)	(0.622)
day -1	0.617***	0.423***	2.294***	1.561**
	(0.116)	(0.093)	(0.758)	(0.625)
day 1	0.688***	0.487***	2.260***	1.537**
	(0.136)	(0.113)	(0.797)	(0.649)
day 2	0.614***	0.414***	2.060***	1.366**
	(0.139)	(0.115)	(0.743)	(0.608)
day 3	0.508***	0.299**	1.956**	1.243*
	(0.148)	(0.126)	(0.815)	(0.658)
day 4	0.523***	0.328***	1.701**	1.068
	(0.142)	(0.119)	(0.810)	(0.646)
day 5	0.494***	0.323**	1.384*	0.863
	(0.146)	(0.127)	(0.733)	(0.606)
day 6	0.432***	0.256**	1.186*	0.648
	(0.141)	(0.124)	(0.703)	(0.560)
day 7	0.272*	0.132	0.934	0.552
	(0.143)	(0.118)	(0.734)	(0.569)
day 8	0.426***	0.254**	1.068	0.562
	(0.139)	(0.124)	(0.693)	(0.526)
day 9	0.452***	0.282**	0.925	0.447
	(0.135)	(0.124)	(0.587)	(0.438)
day 10	0.390**	0.219	0.833	0.344
	(0.149)	(0.132)	(0.600)	(0.455)
day 11	0.392**	0.228*	0.724	0.298
	(0.155)	(0.131)	(0.646)	(0.495)
day 12	0.491***	0.360**	0.388	0.137

	(0.160)	(0.134)	(0.596)	(0.479)
day 13	0.367**	0.241*	0.137	−0.073
	(0.149)	(0.120)	(0.615)	(0.510)
day 14	0.326**	0.236*	−0.085	−0.146
	(0.154)	(0.120)	(0.657)	(0.537)
day 15	0.259*	0.138	0.152	−0.066
	(0.135)	(0.104)	(0.574)	(0.485)
day 16	0.312**	0.175*	0.108	−0.162
	(0.140)	(0.104)	(0.562)	(0.478)
day 17	0.331**	0.207*	0.146	−0.067
	(0.148)	(0.109)	(0.547)	(0.455)
day 18	0.318*	0.209	0.033	−0.138
	(0.162)	(0.141)	(0.432)	(0.391)
day 19	0.196	0.094	−0.038	−0.208
	(0.151)	(0.124)	(0.485)	(0.414)
day 20	0.276*	0.148	0.130	−0.180
	(0.161)	(0.132)	(0.447)	(0.391)
day 21	0.257	0.163	−0.257	−0.383
	(0.156)	(0.126)	(0.404)	(0.384)
day 22	0.270*	0.148	0.188	−0.112
	(0.150)	(0.123)	(0.451)	(0.395)
day 23	0.272*	0.145	0.104	−0.244
	(0.148)	(0.120)	(0.421)	(0.378)
day 24	0.210	0.112	−0.060	−0.300
	(0.140)	(0.114)	(0.441)	(0.390)
day 25	0.198	0.090	0.289	0.017
	(0.138)	(0.114)	(0.449)	(0.407)
day 26	0.129	0.072	0.139	0.049
	(0.127)	(0.115)	(0.424)	(0.419)
day 27	0.169	0.128	0.116	0.113
	(0.124)	(0.105)	(0.371)	(0.369)

day 28	0.141	0.102	0.188	0.202
	(0.117)	(0.099)	(0.415)	(0.374)
day 29	0.257**	0.208**	0.560	0.546
	(0.113)	(0.101)	(0.430)	(0.381)
day 30	0.189*	0.150	0.489	0.495
	(0.110)	(0.102)	(0.400)	(0.374)
day 31	0.187*	0.139	0.428	0.388
	(0.104)	(0.095)	(0.352)	(0.355)
day 32	0.194*	0.143	0.445	0.375
	(0.103)	(0.093)	(0.344)	(0.345)
day 33	0.147	0.132	0.252	0.338
	(0.096)	(0.079)	(0.346)	(0.363)
day 34	0.157*	0.151*	0.268	0.384
	(0.093)	(0.078)	(0.347)	(0.369)
day 35	0.101	0.098	0.202	0.299
	(0.097)	(0.090)	(0.357)	(0.387)
day 36	0.116	0.095	0.309	0.337
	(0.085)	(0.071)	(0.369)	(0.370)
day 37	0.113	0.126*	0.100	0.284
	(0.081)	(0.071)	(0.347)	(0.361)
day 38	0.072	0.107	0.105	0.382
	(0.087)	(0.076)	(0.320)	(0.327)
day 39	0.092	0.112	0.016	0.255
	(0.087)	(0.082)	(0.330)	(0.338)
day 40	0.098	0.134	−0.089	0.213
	(0.086)	(0.085)	(0.307)	(0.340)
Constant	13.914***	0.155	157.262***	102.532***
	(4.614)	(4.280)	(31.907)	(26.332)
Observations	10,078	10,078	10,078	10,078
R^2	0.543	0.630	0.702	0.776
Adjusted R^2	0.537	0.625	0.698	0.773

Residual Std. Error	0.678 (df = 9956)	0.610 (df = 9955)	2.675 (df = 9956)	2.318 (df = 9955)

Note: *p<0.1; **p<0.05; ***p<0.01

S8 Table. Regression table. Event Study regression summary statistics for robustness check (S1 Fig) where we **do not** control for past outcome values (with a delay of 14 days) and growth rates as a way to minimize confounding due to peoples private behavioral changes to COVID-19 [35].

	Dependent variable:		
	confirmed cases	deaths	hospitalization
	(1)	(2)	(3)
new test rate	63.679***	6.182	165.527**
	(19.620)	(13.231)	(63.288)
precipitation	−0.002	−0.003**	0.003
	(0.001)	(0.001)	(0.007)
temperature	0.010*	−0.004	0.016
	(0.005)	(0.006)	(0.022)
retail and recreation	−7.494**	1.258	−34.017*
	(3.153)	(2.832)	(19.747)
grocery and pharmacy	3.617	−4.518	14.073
	(3.774)	(3.380)	(23.450)
parks	0.527***	0.352**	1.811***
	(0.133)	(0.135)	(0.509)
transit stations	−0.898	0.290	−5.671
	(0.780)	(0.659)	(3.489)
workplaces	−1.380	−0.387	6.578
	(1.045)	(0.921)	(5.551)
residential	−67.735***	−41.543***	−382.215***
	(11.767)	(11.082)	(74.552)
grocery and pharmacy squared	−1.807	1.915	−6.424
	(1.681)	(1.518)	(10.253)
retail and recreation squared	3.545**	−1.475	14.954
	(1.713)	(1.525)	(10.426)
parks squared	−0.133***	−0.062**	−0.346***
	(0.028)	(0.027)	(0.105)
transit stations squared	0.739	−0.171	3.364*
	(0.517)	(0.445)	(1.897)

workplaces squared	0.581	0.245	−7.750**
	(0.725)	(0.643)	(3.735)
residential squared	31.377***	20.090***	176.199***
	(5.201)	(4.956)	(33.936)
stay-at-home day	0.0001***	0.0001***	0.0002*
	(0.00003)	(0.00003)	(0.0001)
business closure day	−0.00001	0.0001***	−0.0003**
	(0.00003)	(0.00003)	(0.0001)
day -10	0.470***	0.656***	2.536***
	(0.135)	(0.148)	(0.764)
day -9	0.395***	0.638***	2.338***
	(0.127)	(0.155)	(0.744)
day -8	0.459***	0.676***	2.502***
	(0.137)	(0.171)	(0.761)
day -7	0.441***	0.725***	2.363***
	(0.121)	(0.155)	(0.725)
day -6	0.527***	0.784***	2.890***
	(0.131)	(0.158)	(0.787)
day -5	0.501***	0.788***	2.757***
	(0.120)	(0.158)	(0.758)
day -4	0.547***	0.853***	3.009***
	(0.134)	(0.146)	(0.754)
day -3	0.588***	0.933***	3.588***
	(0.136)	(0.149)	(0.854)
day -2	0.551***	0.856***	3.460***
	(0.129)	(0.140)	(0.835)
day -1	0.577***	0.933***	3.663***
	(0.137)	(0.150)	(0.832)
day 1	0.620***	1.078***	3.955***
	(0.146)	(0.168)	(0.839)

day 2	0.623***	1.026***	3.880***
	(0.149)	(0.170)	(0.777)
day 3	0.664***	0.977***	3.985***
	(0.153)	(0.173)	(0.846)
day 4	0.632***	0.996***	3.787***
	(0.160)	(0.168)	(0.871)
day 5	0.567***	0.947***	3.404***
	(0.146)	(0.166)	(0.750)
day 6	0.581***	0.897***	3.250***
	(0.145)	(0.166)	(0.766)
day 7	0.513***	0.796***	3.065***
	(0.153)	(0.162)	(0.792)
day 8	0.586***	0.933***	3.273***
	(0.151)	(0.162)	(0.780)
day 9	0.578***	0.945***	3.173***
	(0.143)	(0.164)	(0.716)
day 10	0.603***	0.949***	3.208***
	(0.148)	(0.170)	(0.780)
day 11	0.586***	0.956***	3.202***
	(0.157)	(0.172)	(0.864)
day 12	0.492***	1.011***	2.827***
	(0.142)	(0.168)	(0.751)
day 13	0.490***	0.924***	2.704***
	(0.145)	(0.168)	(0.770)
day 14	0.399**	0.862***	2.373***
	(0.163)	(0.181)	(0.868)
day 15	0.496***	0.865***	2.731***
	(0.147)	(0.161)	(0.811)
day 16	0.522***	0.886***	2.690***
	(0.156)	(0.178)	(0.815)
day 17	0.487***	0.888***	2.681***

	(0.169)	(0.176)	(0.856)
day 18	0.454***	0.888***	2.492***
	(0.162)	(0.184)	(0.776)
day 19	0.432**	0.755***	2.274***
	(0.174)	(0.174)	(0.759)
day 20	0.479***	0.778***	2.282***
	(0.170)	(0.187)	(0.765)
day 21	0.386**	0.738***	1.895**
	(0.177)	(0.177)	(0.708)
day 22	0.469***	0.791***	2.308***
	(0.171)	(0.167)	(0.745)
day 23	0.484***	0.800***	2.165***
	(0.165)	(0.171)	(0.700)
day 24	0.403**	0.704***	1.822**
	(0.164)	(0.175)	(0.752)
day 25	0.435**	0.722***	2.276***
	(0.166)	(0.171)	(0.806)
day 26	0.317**	0.668***	1.955**
	(0.144)	(0.155)	(0.737)
day 27	0.261*	0.658***	1.850***
	(0.134)	(0.158)	(0.675)
day 28	0.256*	0.629***	1.942**
	(0.135)	(0.155)	(0.727)
day 29	0.259**	0.677***	2.238***
	(0.128)	(0.145)	(0.783)
day 30	0.239*	0.620***	2.103***
	(0.120)	(0.134)	(0.723)
day 31	0.269**	0.643***	2.091***
	(0.133)	(0.141)	(0.715)
day 32	0.265*	0.620***	1.977***
	(0.133)	(0.149)	(0.710)

day 33	0.163	0.528***	1.626***
	(0.127)	(0.136)	(0.596)
day 34	0.143	0.549***	1.610**
	(0.123)	(0.135)	(0.609)
day 35	0.143	0.510***	1.476**
	(0.121)	(0.140)	(0.574)
day 36	0.173	0.485***	1.554**
	(0.125)	(0.136)	(0.603)
day 37	0.081	0.457***	1.255**
	(0.108)	(0.133)	(0.553)
day 38	0.031	0.420***	1.240**
	(0.109)	(0.139)	(0.558)
day 39	0.066	0.441***	1.255**
	(0.118)	(0.139)	(0.581)
day 40	0.015	0.413***	1.022**
	(0.099)	(0.132)	(0.478)
Constant	36.231***	21.931***	216.114***
	(7.200)	(6.593)	(45.786)
Observations	10,078	10,078	10,078
R^2	0.462	0.336	0.528
Adjusted R^2	0.455	0.328	0.523
Residual Std. Error (df = 9958)	0.737	0.817	3.365

Note: *p<0.1; **p<0.05; ***p<0.01

S9 Table. Regression table. Event Study regression summary statistics of fig. 3 (for earlier states only, i.e. NY, NJ, MA, CT, MI, DC, RI, IL, WA, PA, GA, VT, MD, FL, LA).

	Dependent variable:		
	confirmed cases	deaths	hospitalization
	(1)	(2)	(3)
confirmed cases delay	0.440***		
	(0.048)		
confirmed cases growth	−0.00003		
	(0.0003)		
deaths delay		0.446***	
		(0.040)	
deaths growth		0.001***	
		(0.00005)	
hospitalization delay			0.484***
			(0.056)
hospitalization growth			−0.006
			(0.057)
new test rate	70.321**	20.775*	196.094**
	(25.290)	(11.778)	(87.126)
precipitation	0.001	−0.002	0.004
	(0.001)	(0.001)	(0.010)
temperature	0.008	−0.003	−0.008
	(0.005)	(0.003)	(0.026)
retail and recreation	−10.779***	−4.643**	−61.478***
	(1.638)	(1.733)	(14.706)
grocery and pharmacy	2.930	1.205	27.626
	(3.220)	(2.430)	(23.367)
parks	0.558***	0.293***	1.744
	(0.151)	(0.092)	(1.205)
transit stations	−2.959***	−0.614**	−9.308
	(0.876)	(0.262)	(5.784)

workplaces	3.135**	2.219***	20.317**
	(1.271)	(0.642)	(8.144)
residential	−37.952***	−16.756*	−297.310***
	(9.857)	(9.384)	(70.255)
grocery and pharmacy squared	−1.515	−0.877	−12.125
	(1.632)	(1.225)	(10.741)
retail and recreation squared	5.169***	1.941*	29.777***
	(0.898)	(0.973)	(7.947)
parks squared	−0.089***	−0.039	−0.253
	(0.029)	(0.026)	(0.197)
transit stations squared	2.348**	0.252	5.579
	(0.847)	(0.261)	(3.494)
workplaces squared	−2.546**	−1.326**	−16.194***
	(0.913)	(0.455)	(5.190)
residential squared	18.338***	8.881**	138.113***
	(4.305)	(4.090)	(31.761)
stay-at-home day	0.064**	0.030*	0.280
	(0.026)	(0.016)	(0.199)
business closure day	−0.005	0.087***	0.071
	(0.028)	(0.017)	(0.201)
day -10	0.335	0.379	2.329*
	(0.279)	(0.274)	(1.311)
day -9	0.268	0.282	1.337
	(0.281)	(0.268)	(1.485)
day -8	0.331	0.367	1.470
	(0.265)	(0.283)	(1.294)
day -7	0.366	0.482*	1.157
	(0.252)	(0.249)	(1.281)
day -6	0.321	0.400	1.191
	(0.249)	(0.270)	(1.515)
day -5	0.028	0.297	0.170

	(0.290)	(0.282)	(1.584)
day -4	0.419	0.497*	1.841
	(0.290)	(0.244)	(1.330)
day -3	0.413	0.530**	2.339*
	(0.257)	(0.225)	(1.321)
day -2	0.343	0.370	2.202
	(0.236)	(0.212)	(1.416)
day -1	0.447*	0.486**	2.608*
	(0.250)	(0.209)	(1.274)
day 1	0.283	0.628**	2.565*
	(0.263)	(0.246)	(1.440)
day 2	0.249	0.619**	2.242
	(0.282)	(0.270)	(1.458)
day 3	0.292	0.643**	2.524
	(0.281)	(0.244)	(1.684)
day 4	0.263	0.696**	2.124
	(0.303)	(0.242)	(1.684)
day 5	0.085	0.710***	1.392
	(0.318)	(0.227)	(1.676)
day 6	0.115	0.696***	1.437
	(0.311)	(0.226)	(1.686)
day 7	0.120	0.553*	1.548
	(0.323)	(0.259)	(1.825)
day 8	0.263	0.676***	1.698
	(0.304)	(0.223)	(1.737)
day 9	0.122	0.518**	0.849
	(0.294)	(0.224)	(1.543)
day 10	0.198	0.603**	1.341
	(0.256)	(0.250)	(1.515)
day 11	0.267	0.600**	1.555
	(0.238)	(0.261)	(1.635)

day 12	0.144	0.754**	0.541
	(0.247)	(0.313)	(1.632)
day 13	0.154	0.630**	0.370
	(0.223)	(0.287)	(1.608)
day 14	0.178	0.614*	0.543
	(0.222)	(0.310)	(1.672)
day 15	0.174	0.524*	0.331
	(0.213)	(0.293)	(1.503)
day 16	0.208	0.546*	0.179
	(0.211)	(0.263)	(1.484)
day 17	0.307	0.577*	0.434
	(0.223)	(0.271)	(1.413)
day 18	0.188	0.437	−0.276
	(0.187)	(0.269)	(1.134)
day 19	0.182	0.175	−0.496
	(0.253)	(0.261)	(1.290)
day 20	0.209	0.306	−0.291
	(0.218)	(0.273)	(1.269)
day 21	0.166	0.325	−0.804
	(0.218)	(0.273)	(1.076)
day 22	0.189	0.284	−0.297
	(0.218)	(0.281)	(1.272)
day 23	0.114	0.255	−0.703
	(0.181)	(0.263)	(1.153)
day 24	0.100	0.196	−0.482
	(0.208)	(0.262)	(1.194)
day 25	0.116	0.241	0.124
	(0.193)	(0.236)	(1.207)
day 26	−0.012	0.081	−0.312
	(0.143)	(0.182)	(1.047)

day 27	−0.069	0.103	−0.328
	(0.142)	(0.188)	(0.829)
day 28	−0.120	0.025	−0.311
	(0.173)	(0.192)	(0.844)
day 29	−0.032	0.096	0.338
	(0.131)	(0.164)	(0.940)
day 30	−0.100	0.006	0.210
	(0.106)	(0.148)	(0.930)
day 31	−0.051	0.049	0.394
	(0.125)	(0.154)	(0.835)
day 32	−0.034	0.047	0.379
	(0.120)	(0.148)	(0.774)
day 33	−0.161	0.050	−0.270
	(0.138)	(0.148)	(0.705)
day 34	−0.120	0.154	−0.176
	(0.116)	(0.162)	(0.730)
day 35	−0.129	0.043	−0.488
	(0.143)	(0.122)	(0.628)
day 36	−0.049	0.104	−0.329
	(0.150)	(0.110)	(0.737)
day 37	−0.137	0.024	−0.676
	(0.122)	(0.112)	(0.719)
day 38	−0.205*	−0.007	−0.314
	(0.108)	(0.104)	(0.600)
day 39	−0.132	0.060	−0.319
	(0.093)	(0.111)	(0.579)
day 40	−0.140	0.043	−0.574
	(0.091)	(0.119)	(0.640)
Constant	30.186***	25.208***	220.637***
	(8.618)	(7.743)	(47.945)
Observations	3,164	3,164	3,164

R^2	0.619	0.660	0.774
Adjusted R^2	0.609	0.651	0.767
Residual Std. Error (df = 3078)	0.624	0.589	3.065
Note:			*p<0.1; **p<0.05; ***p<0.01

S10 Table. Regression table. Event Study regression summary statistics of fig. 3 (for later states only, i.e. all states except NY, NJ, MA, CT, MI, DC, RI, IL, WA, PA, GA, VT, MD, FL, LA).

	Dependent variable:		
	confirmed cases	deaths	hospitalization
	(1)	(2)	(3)
confirmed cases delay	0.435^{***}<br>(0.037)		
confirmed cases growth	0.00000<br>(0.00002)		
deaths delay		0.473^{***}<br>(0.041)	
deaths growth		0.0003<br>(0.0003)	
hospitalization delay			0.491^{***}<br>(0.065)
hospitalization growth			0.134^{***}<br>(0.038)
new test rate	64.443^{***}<br>(19.220)	40.579^{**}<br>(15.755)	152.186^{***}<br>(50.397)
precipitation	-0.004^{**}<br>(0.001)	-0.007^{***}<br>(0.002)	-0.008<br>(0.006)
temperature	0.018^{***}<br>(0.004)	0.006<br>(0.006)	0.082^{***}<br>(0.015)
retail and recreation	-1.804<br>(1.946)	2.654<br>(1.581)	-0.137<br>(7.024)
grocery and pharmacy	3.598<br>(2.720)	-3.414<br>(2.406)	-9.419<br>(12.807)
parks	0.294^{**}<br>(0.110)	0.235^{***}<br>(0.084)	0.785^{***}<br>(0.238)
transit stations	-0.622<br>(0.473)	0.351<br>(0.513)	-1.551<br>(1.783)

workplaces	1.581	2.478***	8.994***
	(0.966)	(0.829)	(3.228)
residential	−23.420	−28.435**	−154.735**
	(14.672)	(12.870)	(63.285)
grocery and pharmacy squared	−1.959	1.178	2.844
	(1.229)	(1.062)	(5.674)
retail and recreation squared	0.360	−2.322***	−1.976
	(1.138)	(0.849)	(3.731)
parks squared	−0.069***	−0.037**	−0.138**
	(0.023)	(0.018)	(0.053)
transit stations squared	0.595*	0.009	1.473
	(0.320)	(0.301)	(1.161)
workplaces squared	−1.467**	−1.803***	−8.096***
	(0.702)	(0.635)	(2.287)
residential squared	11.549*	14.558**	73.529**
	(6.592)	(5.815)	(28.528)
stay-at-home day	0.00004	0.00004	−0.0001
	(0.00003)	(0.00003)	(0.0001)
business closure day	0.0001**	0.0001***	0.00004
	(0.00003)	(0.00003)	(0.0001)
day -10	0.298***	0.480***	1.080*
	(0.100)	(0.128)	(0.552)
day -9	0.264**	0.544***	1.324**
	(0.100)	(0.147)	(0.601)
day -8	0.286***	0.495***	1.148*
	(0.100)	(0.169)	(0.588)
day -7	0.228**	0.519***	1.047*
	(0.095)	(0.151)	(0.596)
day -6	0.321***	0.590***	1.446*
	(0.110)	(0.143)	(0.715)

day -5	0.379***	0.647***	1.576**
	(0.108)	(0.138)	(0.712)
day -4	0.275**	0.591***	1.254*
	(0.105)	(0.134)	(0.703)
day -3	0.286**	0.652***	1.612*
	(0.124)	(0.148)	(0.878)
day -2	0.351***	0.674***	1.836**
	(0.128)	(0.127)	(0.890)
day -1	0.301**	0.649***	1.701*
	(0.121)	(0.146)	(0.864)
day 1	0.347**	0.687***	1.665*
	(0.143)	(0.170)	(0.901)
day 2	0.375**	0.597***	1.547*
	(0.143)	(0.172)	(0.809)
day 3	0.337**	0.403**	1.191
	(0.144)	(0.191)	(0.779)
day 4	0.235	0.387**	0.984
	(0.141)	(0.175)	(0.796)
day 5	0.306**	0.375*	0.977
	(0.138)	(0.187)	(0.712)
day 6	0.272**	0.271	0.586
	(0.129)	(0.169)	(0.615)
day 7	0.141	0.103	0.236
	(0.120)	(0.159)	(0.550)
day 8	0.165	0.275	0.397
	(0.117)	(0.168)	(0.473)
day 9	0.221*	0.395**	0.464
	(0.116)	(0.170)	(0.385)
day 10	0.149	0.249	0.135
	(0.111)	(0.179)	(0.365)
day 11	0.100	0.250	−0.131

	(0.130)	(0.185)	(0.417)
day 12	0.121	0.362*	−0.017
	(0.112)	(0.179)	(0.391)
day 13	0.083	0.205	−0.347
	(0.110)	(0.159)	(0.440)
day 14	−0.050	0.123	−0.699
	(0.105)	(0.154)	(0.471)
day 15	0.077	0.116	−0.289
	(0.106)	(0.127)	(0.429)
day 16	0.083	0.169	−0.301
	(0.128)	(0.150)	(0.433)
day 17	−0.031	0.183	−0.297
	(0.116)	(0.155)	(0.449)
day 18	−0.038	0.236	−0.275
	(0.123)	(0.194)	(0.369)
day 19	0.009	0.207	−0.150
	(0.137)	(0.165)	(0.376)
day 20	0.064	0.252	−0.081
	(0.129)	(0.180)	(0.322)
day 21	−0.020	0.198	−0.384
	(0.147)	(0.170)	(0.303)
day 22	0.092	0.249	−0.007
	(0.135)	(0.162)	(0.305)
day 23	0.173	0.289	0.197
	(0.160)	(0.177)	(0.348)
day 24	0.069	0.208	−0.215
	(0.125)	(0.157)	(0.372)
day 25	0.048	0.156	−0.059
	(0.129)	(0.170)	(0.449)
day 26	0.064	0.162	0.019
	(0.119)	(0.162)	(0.499)

day 27	0.015	0.193	−0.054
	(0.105)	(0.155)	(0.464)
day 28	0.001	0.190	0.039
	(0.088)	(0.137)	(0.524)
day 29	0.026	0.339**	0.369
	(0.080)	(0.137)	(0.484)
day 30	0.062	0.303**	0.395
	(0.102)	(0.138)	(0.417)
day 31	0.039	0.258**	0.203
	(0.105)	(0.127)	(0.373)
day 32	0.026	0.264**	0.213
	(0.097)	(0.129)	(0.392)
day 33	−0.003	0.218*	0.366
	(0.098)	(0.125)	(0.413)
day 34	−0.071	0.154	0.277
	(0.085)	(0.120)	(0.405)
day 35	−0.114	0.114	0.224
	(0.079)	(0.133)	(0.457)
day 36	−0.041	0.108	0.348
	(0.098)	(0.121)	(0.439)
day 37	−0.107	0.161	0.255
	(0.084)	(0.112)	(0.417)
day 38	−0.157*	0.113	0.176
	(0.082)	(0.121)	(0.378)
day 39	−0.164*	0.096	0.037
	(0.081)	(0.122)	(0.399)
day 40	−0.100	0.135	0.137
	(0.080)	(0.115)	(0.339)
Constant	8.872	12.541*	84.933**
	(8.803)	(7.334)	(39.655)

Observations	6,914	6,914	6,914
R^2	0.687	0.525	0.631
Adjusted R^2	0.682	0.518	0.626
Residual Std. Error (df = 6807)	0.563	0.692	2.316
Note:			*p<0.1; **p<0.05; ***p<0.01

S11 Table. Regression table. Event Study regression summary statistics for robustness check (S4. Fig) where we focus on county-level mandates only.

	Dependent variable:		
	confirmed cases	deaths	hospitalization
	(1)	(2)	(3)
confirmed cases delay	0.195***		
	(0.003)		
confirmed cases growth	−0.000***		
	(0.000)		
deaths delay		0.144***	
		(0.003)	
deaths growth		0.000*	
		(0.000)	
hospitalization delay			0.456***
			(0.003)
hospitalization growth			0.252***
			(0.002)
new test rate	70.678***	33.179***	158.236***
	(3.122)	(3.772)	(19.696)
precipitation	0.0005	−0.0003	0.007**
	(0.0005)	(0.001)	(0.003)
temperature	−0.019***	−0.015***	−0.029***
	(0.001)	(0.001)	(0.003)
retail and recreation	−0.080	0.030	−1.973***
	(0.063)	(0.076)	(0.398)
grocery and pharmacy	−0.256***	−0.058	2.877***
	(0.055)	(0.067)	(0.349)
parks	0.095***	0.044***	0.030
	(0.009)	(0.011)	(0.059)
transit stations	−0.060**	−0.052*	0.716***
	(0.024)	(0.029)	(0.151)

workplaces	−0.209*	−0.187	−2.603***
	(0.120)	(0.145)	(0.756)
residential	−1.432***	−1.209***	−15.006***
	(0.096)	(0.116)	(0.606)
grocery and pharmacy squared	0.212***	0.079	−2.125***
	(0.043)	(0.052)	(0.273)
retail and recreation squared	−0.153***	−0.120*	0.809**
	(0.053)	(0.064)	(0.332)
parks squared	−0.039***	−0.020***	−0.088***
	(0.004)	(0.004)	(0.023)
transit stations squared	0.051**	0.033	−0.162
	(0.020)	(0.024)	(0.128)
workplaces squared	0.098	0.115	3.676***
	(0.108)	(0.130)	(0.681)
residential squared	1.394***	1.149***	13.860***
	(0.088)	(0.107)	(0.557)
stay-at-home day	0.00001***	0.00000	−0.00003***
	(0.00000)	(0.00000)	(0.00001)
business closure day	0.00000	0.00002	−0.002***
	(0.00002)	(0.00002)	(0.0001)
day -10	0.330***	0.135***	0.700***
	(0.037)	(0.045)	(0.234)
day -9	0.357***	0.069	0.732***
	(0.037)	(0.045)	(0.235)
day -8	0.372***	0.085*	0.722***
	(0.037)	(0.045)	(0.232)
day -7	0.411***	0.126***	0.763***
	(0.037)	(0.044)	(0.232)
day -6	0.463***	0.187***	0.864***
	(0.037)	(0.045)	(0.232)
day -5	0.517***	0.219***	0.875***

	(0.037)	(0.044)	(0.232)
day -4	0.572***	0.305***	0.947***
	(0.037)	(0.044)	(0.232)
day -3	0.634***	0.284***	1.048***
	(0.037)	(0.045)	(0.234)
day -2	0.673***	0.365***	1.121***
	(0.037)	(0.045)	(0.235)
day -1	0.719***	0.443***	1.176***
	(0.038)	(0.045)	(0.237)
day 1	0.492***	0.177***	1.027***
	(0.048)	(0.058)	(0.303)
day 2	0.440***	0.140**	1.152***
	(0.048)	(0.058)	(0.303)
day 3	0.406***	0.192***	1.300***
	(0.048)	(0.058)	(0.304)
day 4	0.437***	0.261***	1.517***
	(0.048)	(0.058)	(0.303)
day 5	0.446***	0.214***	1.460***
	(0.048)	(0.058)	(0.302)
day 6	0.499***	0.226***	1.688***
	(0.049)	(0.059)	(0.308)
day 7	0.480***	0.251***	1.732***
	(0.049)	(0.059)	(0.308)
day 8	0.472***	0.244***	1.886***
	(0.049)	(0.059)	(0.309)
day 9	0.471***	0.261***	1.713***
	(0.050)	(0.060)	(0.314)
day 10	0.482***	0.264***	1.692***
	(0.050)	(0.061)	(0.316)
day 11	0.454***	0.269***	1.764***
	(0.051)	(0.061)	(0.319)

day 12	0.441***	0.289***	1.919***
	(0.050)	(0.061)	(0.317)
day 13	0.445***	0.322***	2.027***
	(0.051)	(0.062)	(0.322)
day 14	0.415***	0.297***	1.877***
	(0.051)	(0.061)	(0.320)
day 15	0.385***	0.264***	1.754***
	(0.051)	(0.062)	(0.323)
day 16	0.312***	0.274***	1.693***
	(0.051)	(0.062)	(0.324)
day 17	0.294***	0.283***	1.656***
	(0.052)	(0.062)	(0.326)
day 18	0.329***	0.323***	1.664***
	(0.052)	(0.063)	(0.329)
day 19	0.274***	0.318***	1.683***
	(0.052)	(0.063)	(0.329)
day 20	0.251***	0.302***	1.632***
	(0.052)	(0.063)	(0.330)
day 21	0.224***	0.348***	1.298***
	(0.053)	(0.064)	(0.332)
day 22	0.137***	0.355***	1.131***
	(0.053)	(0.064)	(0.332)
day 23	0.119**	0.264***	1.128***
	(0.053)	(0.064)	(0.335)
day 24	0.056	0.278***	1.249***
	(0.053)	(0.064)	(0.336)
day 25	0.042	0.463***	1.102***
	(0.054)	(0.065)	(0.338)
day 26	0.061	0.474***	0.922***
	(0.054)	(0.065)	(0.340)

day 27	0.022	0.542***	0.727**
	(0.054)	(0.065)	(0.339)
day 28	−0.024	0.462***	0.594*
	(0.054)	(0.065)	(0.340)
day 29	−0.010	0.488***	0.475
	(0.055)	(0.066)	(0.345)
day 30	−0.095*	0.544***	0.426
	(0.055)	(0.067)	(0.348)
day 31	−0.060	0.416***	0.680*
	(0.056)	(0.067)	(0.350)
day 32	−0.088	0.185***	0.472
	(0.056)	(0.067)	(0.351)
day 33	−0.085	0.123*	0.385
	(0.056)	(0.067)	(0.350)
day 34	−0.070	0.121*	0.533
	(0.055)	(0.067)	(0.347)
day 35	−0.081	0.230***	0.389
	(0.056)	(0.067)	(0.351)
day 36	−0.080	0.270***	0.323
	(0.055)	(0.067)	(0.350)
day 37	−0.023	0.291***	0.303
	(0.056)	(0.068)	(0.356)
day 38	−0.014	0.313***	0.104
	(0.056)	(0.068)	(0.353)
day 39	−0.018	0.259***	0.076
	(0.056)	(0.068)	(0.354)
day 40	0.059	0.221***	0.113
	(0.057)	(0.069)	(0.358)
day 41	0.079	0.256***	0.012
	(0.058)	(0.070)	(0.365)
day 42	−0.031	0.129*	−0.267

	(0.058)	(0.070)	(0.367)
day 43	0.064	0.189***	−0.218
	(0.058)	(0.070)	(0.366)
day 44	0.068	0.189***	−0.319
	(0.058)	(0.070)	(0.364)
day 45	0.017	0.190***	−0.316
	(0.059)	(0.071)	(0.370)
day 46	0.027	0.232***	−0.221
	(0.058)	(0.071)	(0.369)
day 47	0.021	0.238***	−0.014
	(0.060)	(0.072)	(0.376)
day 48	0.030	0.226***	−0.118
	(0.060)	(0.072)	(0.376)
day 49	−0.028	0.212***	−0.047
	(0.059)	(0.072)	(0.373)
day 50	−0.015	0.126*	−0.154
	(0.060)	(0.072)	(0.376)
day 51	−0.071	0.080	−0.299
	(0.061)	(0.074)	(0.385)
day 52	−0.090	0.089	−0.173
	(0.061)	(0.074)	(0.386)
day 53	−0.019	0.102	−0.122
	(0.061)	(0.073)	(0.383)
day 54	−0.065	0.123	−0.065
	(0.062)	(0.075)	(0.389)
day 55	−0.033	0.099	−0.067
	(0.062)	(0.074)	(0.388)
day 56	−0.019	0.045	0.034
	(0.062)	(0.075)	(0.390)
day 57	−0.059	0.055	0.004
	(0.063)	(0.076)	(0.399)

day 58	−0.105	0.001	−0.030
	(0.064)	(0.078)	(0.406)
day 59	−0.113*	0.028	−0.075
	(0.064)	(0.077)	(0.404)
day 60	−0.081	0.008	0.023
	(0.065)	(0.079)	(0.411)
day 61	−0.108	0.035	−0.200
	(0.066)	(0.080)	(0.418)
day 62	−0.130*	0.014	−0.274
	(0.067)	(0.080)	(0.420)
day 63	−0.157**	−0.057	−0.184
	(0.068)	(0.082)	(0.427)
day 64	−0.138**	−0.115	−0.314
	(0.069)	(0.083)	(0.433)
day 65	−0.147**	−0.130	−0.407
	(0.070)	(0.084)	(0.441)
day 66	−0.179**	−0.239***	−0.345
	(0.070)	(0.084)	(0.441)
day 67	−0.149**	−0.178**	−0.327
	(0.070)	(0.085)	(0.443)
day 68	−0.174**	−0.095	−0.477
	(0.070)	(0.085)	(0.443)
day 69	−0.034	−0.157*	−0.345
	(0.071)	(0.086)	(0.450)
day 70	−0.114	−0.376***	−0.244
	(0.072)	(0.087)	(0.454)
Observations	77,930	77,930	77,930
R^2	0.502	0.272	0.469
Adjusted R^2	0.500	0.269	0.466
Residual Std. Error (df = 77610)	0.704	0.851	4.441
Note:			*p<0.1; **p<0.05; ***p<0.01

S12 Table. Regression table. Event Study regression summary statistics for robustness check (fig. 6) where we estimate the effect of lifting mask mandates on COVID-19 outcomes.

	Dependent variable:		
	confirmed cases	deaths	hospitalization
	(1)	(2)	(3)
confirmed cases delay 14days	0.613***		
	(0.017)		
confirmed cases delay 14days growth	0.00002		
	(0.0001)		
deaths delay 14days		0.575***	
		(0.028)	
deaths delay 14days growth		−0.00005	
		(0.0002)	
hospitalization delay 14days			0.654***
			(0.017)
hospitalization delay 14days growth			0.186***
			(0.029)
new test rate	14.800***	8.923*	19.825
	(5.457)	(4.646)	(13.762)
precipitation avg	−0.002***	−0.003***	−0.003
	(0.001)	(0.001)	(0.004)
temperature avg	0.001	−0.004***	−0.002
	(0.002)	(0.001)	(0.008)
people vaccinated per hundred	−0.048***	−0.039***	−0.160***
	(0.004)	(0.004)	(0.016)
retail and recreation	−3.840***	−3.348***	−18.619***
	(0.452)	(1.062)	(5.916)
grocery and pharmacy	5.101***	3.738***	17.709**
	(0.586)	(1.267)	(6.999)
parks	0.114***	0.143***	0.809***
	(0.029)	(0.046)	(0.247)

transit stations	-0.478^{**}	-0.714^{*}	-1.843
	(0.234)	(0.382)	(1.436)
workplaces	1.537^{***}	3.123^{***}	9.226^{***}
	(0.369)	(0.569)	(3.102)
residential	12.729^{**}	-10.692	-73.815
	(5.709)	(8.634)	(44.545)
grocery and pharmacy square	-2.118^{***}	-1.369^{**}	-7.170^{**}
	(0.264)	(0.562)	(3.107)
retail and recreation square	1.814^{***}	1.350^{**}	8.810^{***}
	(0.252)	(0.580)	(3.152)
parks square	-0.042^{***}	-0.025^{**}	-0.244^{***}
	(0.007)	(0.010)	(0.058)
transit stations square	0.453^{***}	0.183	1.284
	(0.153)	(0.207)	(0.837)
workplaces square	-0.397	-1.272^{***}	-5.457^{**}
	(0.244)	(0.411)	(2.085)
residential square	-4.168	6.969^{*}	38.576^{*}
	(2.614)	(3.864)	(20.154)
stay-at-home day	-0.00000	0.00002^{**}	0.0001^{*}
	(0.00001)	(0.00001)	(0.00003)
business closure day	-0.00002	-0.00001	-0.0001^{***}
	(0.00001)	(0.00001)	(0.00004)
day -45	-0.468^{***}	0.103	-0.873
	(0.157)	(0.255)	(0.525)
day -44	-0.487^{***}	-0.292	-1.350^{***}
	(0.151)	(0.316)	(0.454)
day -43	-0.742^{***}	-0.524	-0.973
	(0.219)	(0.388)	(1.100)
day -42	-0.673^{***}	-0.494	-0.530
	(0.193)	(0.387)	(0.990)
day -41	-0.730^{***}	-0.670	-0.850

	(0.217)	(0.410)	(1.136)
day -40	-0.658^{***}	-0.539	-1.013
	(0.217)	(0.546)	(1.194)
day -39	-0.872^{***}	-0.934^{**}	-2.484^{***}
	(0.267)	(0.434)	(0.718)
day -38	-0.725^{**}	-0.846^{*}	-2.182^{***}
	(0.279)	(0.494)	(0.636)
day -37	-0.619^{*}	-0.504^{**}	-2.279^{***}
	(0.329)	(0.234)	(0.673)
day -36	-0.732^{**}	-0.459^{*}	-2.381^{***}
	(0.318)	(0.259)	(0.593)
day -35	-0.183	-0.174	-1.771^{*}
	(0.415)	(0.309)	(0.938)
day -34	-0.572^{**}	-0.319	-2.282^{***}
	(0.252)	(0.243)	(0.717)
day -33	-0.515^{*}	-0.360	-2.294^{**}
	(0.281)	(0.277)	(0.923)
day -32	-0.512^{**}	-0.265	-2.668^{***}
	(0.234)	(0.199)	(0.964)
day -31	-0.476^{*}	-0.379	-2.779^{***}
	(0.239)	(0.265)	(1.025)
day -30	-0.418^{**}	-0.532	-2.664^{**}
	(0.208)	(0.472)	(1.115)
day -29	-0.530^{**}	-0.749^{*}	-3.288^{***}
	(0.244)	(0.410)	(1.023)
day -28	-0.494^{*}	-0.814^{**}	-3.154^{***}
	(0.251)	(0.397)	(1.169)
day -27	-0.500^{*}	-0.961^{**}	-3.353^{**}
	(0.259)	(0.382)	(1.302)
day -26	-0.388	-0.731^{*}	-3.181^{**}
	(0.236)	(0.404)	(1.469)

day -25	-0.287^{*}	-0.715^{*}	-2.200^{***}
	(0.162)	(0.410)	(0.665)
day -24	-0.522^{**}	-0.915^{**}	-2.472^{***}
	(0.256)	(0.375)	(0.619)
day -23	-0.727^{**}	-0.798^{***}	-2.583^{***}
	(0.308)	(0.266)	(0.863)
day -22	-0.641^{***}	-0.742^{***}	-2.424^{***}
	(0.217)	(0.211)	(0.632)
day -21	-0.716^{***}	-0.862^{***}	-2.694^{***}
	(0.235)	(0.287)	(0.654)
day -20	-0.761^{***}	-0.655^{**}	-2.695^{***}
	(0.255)	(0.258)	(0.659)
day -19	-0.711^{**}	-0.609^{*}	-2.182^{***}
	(0.318)	(0.338)	(0.685)
day -18	-0.759^{***}	-0.595^{**}	-1.963^{***}
	(0.255)	(0.266)	(0.500)
day -17	-0.709^{***}	-0.196	-1.902^{***}
	(0.234)	(0.317)	(0.524)
day -16	-0.711^{***}	0.118	-1.954^{***}
	(0.172)	(0.455)	(0.565)
day -15	-0.677^{***}	0.196	-1.943^{***}
	(0.157)	(0.426)	(0.576)
day -14	-0.584^{***}	0.373	-1.889^{***}
	(0.136)	(0.445)	(0.548)
day -13	-0.784^{***}	0.075	-1.302
	(0.278)	(0.345)	(0.845)
day -12	-0.638^{**}	-0.064	-0.821
	(0.273)	(0.322)	(0.866)
day -11	-0.716^{***}	-0.169	-1.029
	(0.252)	(0.340)	(0.722)

day -10	-0.616^{**}	-0.229	-0.958
	(0.263)	(0.322)	(0.630)
day -9	-0.636^{**}	-0.136	-0.980
	(0.264)	(0.287)	(0.637)
day -8	-0.605^{**}	-0.070	-1.001^{*}
	(0.233)	(0.266)	(0.534)
day -7	-0.562^{**}	0.148	-0.918^{**}
	(0.231)	(0.323)	(0.434)
day -6	-0.406^{*}	0.052	-0.833^{*}
	(0.204)	(0.335)	(0.443)
day -5	-0.239	0.108	-0.989^{**}
	(0.224)	(0.403)	(0.417)
day -4	-0.230	0.101	-0.985^{**}
	(0.207)	(0.434)	(0.465)
day -3	-0.272	0.036	-0.970
	(0.203)	(0.351)	(0.699)
day -2	-0.220	-0.254	-0.821
	(0.288)	(0.457)	(0.767)
day -1	-0.457^{***}	-0.125	-0.812
	(0.165)	(0.518)	(0.960)
day 1	-0.380^{**}	-0.413	-0.824
	(0.168)	(0.321)	(0.903)
day 2	-0.527^{**}	-0.331	-0.745
	(0.219)	(0.302)	(0.919)
day 3	-0.603^{***}	-0.099	-0.800
	(0.221)	(0.108)	(1.062)
day 4	-0.452^{***}	-0.204	-0.588
	(0.168)	(0.136)	(1.123)
day 5	-0.557^{***}	-0.539^{**}	-0.537
	(0.155)	(0.268)	(1.024)
day 6	-0.100	-0.611^{***}	-0.503

	(0.329)	(0.191)	(0.973)
day 7	−0.372***	−0.928**	−0.565
	(0.118)	(0.348)	(0.924)
day 8	−0.471***	−0.900**	−1.065
	(0.171)	(0.432)	(1.108)
day 9	−0.345**	−0.798**	−0.863
	(0.157)	(0.376)	(0.802)
day 10	−0.348***	−0.908**	−0.976
	(0.125)	(0.356)	(0.697)
day 11	−0.345***	−0.956**	−1.179**
	(0.119)	(0.382)	(0.548)
day 12	−0.317***	−0.984***	−1.163**
	(0.113)	(0.362)	(0.444)
day 13	−0.316***	−0.946***	−1.179**
	(0.107)	(0.350)	(0.481)
day 14	−0.288***	−0.530**	−1.067**
	(0.102)	(0.211)	(0.523)
day 15	−0.236*	−0.482**	−0.847
	(0.124)	(0.205)	(0.700)
day 16	−0.130	−0.342**	−0.068
	(0.127)	(0.158)	(0.555)
day 17	−0.128	−0.350**	−0.013
	(0.109)	(0.138)	(0.621)
day 18	−0.234*	−0.443*	−0.899
	(0.135)	(0.240)	(1.001)
day 19	−0.231*	−0.463	−0.537
	(0.125)	(0.290)	(1.015)
day 20	−0.216*	−0.303	−0.330
	(0.116)	(0.242)	(0.909)
day 21	−0.198**	−0.245	0.023
	(0.097)	(0.224)	(0.803)

day 22	-0.226^{*}	-0.298	0.301
	(0.120)	(0.210)	(0.766)
day 23	-0.211	-0.228	0.559
	(0.163)	(0.222)	(0.729)
day 24	-0.147	-0.240	0.721
	(0.186)	(0.263)	(0.760)
day 25	-0.248^{*}	-0.449^{**}	0.487
	(0.125)	(0.189)	(0.938)
day 26	-0.196^{**}	-0.316^{**}	0.603
	(0.084)	(0.149)	(0.962)
day 27	-0.148^{**}	-0.291^{**}	0.588
	(0.068)	(0.120)	(0.907)
day 28	-0.335^{*}	-0.493^{**}	-1.063^{***}
	(0.197)	(0.236)	(0.337)
day 29	-0.170^{**}	-0.340^{*}	-0.451^{**}
	(0.079)	(0.189)	(0.182)
day 30	-0.139	-0.340	-0.589^{**}
	(0.115)	(0.232)	(0.259)
day 31	-0.174^{**}	-0.255	-0.834^{**}
	(0.085)	(0.166)	(0.334)
day 32	-0.091^{*}	-0.259^{**}	-0.810^{**}
	(0.053)	(0.113)	(0.345)
day 33	-0.164	-0.268^{***}	-1.020^{*}
	(0.098)	(0.090)	(0.599)
day 34	-0.076	-0.211^{***}	-1.068
	(0.079)	(0.061)	(0.785)
day 35	-0.097	-0.217^{**}	-1.157
	(0.105)	(0.088)	(0.963)
day 36	-0.070	-0.278^{***}	-1.461
	(0.081)	(0.070)	(1.113)

day 37	−0.040	−0.237***	−1.623
	(0.079)	(0.083)	(1.192)
day 38	−0.055	−0.260**	−1.777
	(0.068)	(0.101)	(1.192)
day 39	−0.001	−0.253**	−1.531
	(0.079)	(0.105)	(1.212)
day 40	0.013	−0.248***	−1.285
	(0.078)	(0.076)	(1.027)
day 41	0.079	−0.187**	−0.972
	(0.049)	(0.083)	(0.919)
day 42	0.074	−0.125	−0.811
	(0.053)	(0.125)	(0.829)
day 43	0.035	−0.109	−0.614
	(0.067)	(0.134)	(0.779)
day 44	0.068	−0.160	−0.537
	(0.101)	(0.153)	(0.616)
day 45	0.101	−0.167	−0.403
	(0.075)	(0.149)	(0.407)
Constant	−11.904***	0.838	28.154
	(3.094)	(4.721)	(23.745)
Observations	19,502	19,502	19,502
R^2	0.793	0.657	0.716
Adjusted R^2	0.792	0.654	0.714
Residual Std. Error (df = 19339)	0.456	0.588	2.416
Note:			*p<0.1; **p<0.05; ***p<0.01

S13 Table. Regression table. Event Study regression summary statistics of testing rate event study shown in S2. Fig.

	Dependent variable:
	full formula
precipitation	0.00000
	(0.00000)
temperature	0.00001
	(0.00001)
retail and recreation	-0.003^{*}
	(0.002)
grocery and pharmacy	0.005^{***}
	(0.001)
parks	0.0001
	(0.0001)
transit stations	-0.0005
	(0.001)
workplaces	0.002
	(0.001)
residential	0.038^{**}
	(0.017)
grocery and pharmacy squared	-0.002^{***}
	(0.001)
retail and recreation squared	0.001
	(0.001)
parks squared	-0.00005^{**}
	(0.00002)
transit stations squared	0.001^{*}
	(0.0005)
workplaces squared	-0.001
	(0.001)
residential squared	-0.016^{**}
	(0.007)

day -10	-0.00002<br>(0.0001)
day -9	0.0001<br>(0.0001)
day -8	0.00004<br>(0.0001)
day -7	0.0001<br>(0.0001)
day -6	-0.0001<br>(0.0001)
day -5	-0.0001<br>(0.0001)
day -4	0.00005<br>(0.0001)
day -3	-0.00005<br>(0.0001)
day -2	0.0001^{*}<br>(0.0001)
day -1	-0.0001<br>(0.0001)
day 1	-0.00003<br>(0.0001)
day 2	-0.0001<br>(0.0001)
day 3	-0.0001<br>(0.0001)
day 4	0.0002<br>(0.0001)
day 5	0.0001<br>(0.0001)

day 6	0.0001
	(0.0001)
day 7	0.00004
	(0.0001)
day 8	0.0001
	(0.0001)
day 9	−0.00003
	(0.0001)
day 10	0.00002
	(0.0001)
day 11	0.0002
	(0.0002)
day 12	0.0001
	(0.0001)
day 13	0.0001
	(0.0001)
day 14	0.00002
	(0.0001)
day 15	−0.0001
	(0.0001)
day 16	−0.0001
	(0.0001)
day 17	−0.0001
	(0.0001)
day 18	0.0001
	(0.0002)
day 19	0.0004*
	(0.0002)
day 20	−0.00001
	(0.0001)
day 21	−0.0001

	(0.0001)
day 22	−0.0002
	(0.0001)
day 23	−0.0002*
	(0.0001)
day 24	−0.0002**
	(0.0001)
day 25	−0.0001
	(0.0001)
day 26	0.0001
	(0.0001)
day 27	0.0001
	(0.0001)
day 28	−0.0003**
	(0.0001)
day 29	−0.0001
	(0.0001)
day 30	−0.0002*
	(0.0001)
day 31	−0.0003**
	(0.0001)
day 32	−0.0001
	(0.0001)
day 33	0.0001
	(0.0003)
day 34	0.0001
	(0.0001)
day 35	−0.0002**
	(0.0001)
day 36	−0.0003***
	(0.0001)

day 37	−0.0002
	(0.0001)
day 38	−0.0001
	(0.0001)
day 39	−0.00003
	(0.0001)
day 40	−0.0001
	(0.0001)
Constant	−0.027***
	(0.010)
Observations	10,078
R^2	0.501
Adjusted R^2	0.495
Residual Std. Error	0.001 (df = 9961)
Note:	*p<0.1; **p<0.05; ***p<0.01

S14 Table. Regression table. Event Study regression summary statistics for robustness check comparing controlling for cases, deaths and testing rate (S7. Fig).

	Dependent variable:	
	Mask Adherence (all controls)	Mask Adherence (no cases, deaths and tests)
deaths	7.552	
	(3.519)	
confirmed cases	0.113	
	(0.073)	
new test rate	−25.681	
	(14.831)	
grocery and pharmacy	0.160	0.184
	(0.074)	(0.096)
parks	0.005	0.012
	(0.009)	(0.017)
transit stations	−0.014	−0.028
	(0.030)	(0.055)
workplaces	0.024	0.129
	(0.121)	(0.155)
residential	0.603	1.522*
	(0.388)	(0.587)
grocery and pharmacy square	−0.001	−0.002
	(0.002)	(0.002)
retail and recreation square	0.003**	0.004**
	(0.001)	(0.001)
parks square	0.0001	0.0002*
	(0.00004)	(0.0001)
transit stations square	0.002	0.003*
	(0.001)	(0.001)
workplaces square	−0.003	−0.001
	(0.003)	(0.004)

residential square	−0.010	−0.050
	(0.022)	(0.039)
day -8	−2.754	0.075
	(2.695)	(0.923)
day -7	−4.122	−0.786
	(3.912)	(1.331)
day -6	−3.599	0.447
	(3.670)	(1.733)
day -5	−3.251	0.905
	(4.414)	(1.795)
day -4	−3.774	0.340
	(4.184)	(1.224)
day -3	−3.953	0.706
	(3.944)	(0.890)
day -2	−3.885	−0.341
	(3.836)	(1.161)
day -1	−2.264	0.756
	(2.695)	(0.866)
day 0	−3.137	−0.280
	(3.065)	(1.281)
day 1	−2.550	0.678
	(2.372)	(0.527)
day 2	−3.244	−0.279
	(2.511)	(1.276)
day 3	−2.915	−0.197
	(2.247)	(1.010)
day 4	−2.293	0.936
	(2.602)	(1.573)
day 5	−1.656	1.048
	(1.836)	(1.336)
day 6	−1.150	1.626

	(2.238)	(2.135)
day 7	1.319	3.889
	(1.827)	(2.240)
day 8	2.357	5.019
	(1.220)	(2.431)
day 9	3.295	5.668
	(1.560)	(3.124)
day 10	5.259*	7.243
	(1.716)	(4.020)
day 11	8.908**	10.077*
	(2.656)	(3.937)
day 12	9.934	7.471
	(4.691)	(6.907)
day 13	21.114**	16.732*
	(5.825)	(6.980)
Constant	70.346***	62.648***
	(6.894)	(8.436)
Observations	323	323
R^2	0.934	0.903
Adjusted R^2	0.925	0.890
Residual Std. Error	2.538 (df = 281)	3.070 (df = 284)
Note:		*p<0.1; **p<0.05; ***p<0.01

S15 Table. Regression table. Event Study regression summary statistics for robustness check after mask mandate lifting comparing controlling for cases, deaths and testing rate (S8. Fig).

	Dependent variable:	
	Mask Adherence (all controls)	Mask Adherence (no cases, deaths and tests)
deaths	1.026*** (0.267)	
confirmed cases	0.005 (0.005)	
new test rate	−7.043 (10.196)	
people vaccinated per hundred	0.032 (0.024)	
precipitation avg	−0.006 (0.004)	−0.008* (0.005)
temperature avg	−0.030* (0.016)	−0.028* (0.016)
retail and recreation	8.965 (5.927)	7.067 (6.075)
grocery and pharmacy	−12.881*** (3.582)	−12.197*** (3.744)
parks	2.104** (0.966)	2.492** (1.032)
transit stations	11.585** (5.665)	11.328* (5.947)
workplaces	−16.563* (8.684)	−15.012* (8.908)
residential	413.874*** (74.551)	452.016*** (78.833)
grocery and pharmacy square	4.966** (2.024)	5.559** (2.087)

retail and recreation square	−1.921	−1.250
	(3.303)	(3.322)
parks square	−0.522*	−0.608*
	(0.295)	(0.318)
transit stations square	−9.145**	−9.303**
	(3.427)	(3.588)
workplaces square	15.746***	15.339***
	(5.015)	(5.187)
residential square	−173.674***	−188.029***
	(31.891)	(33.784)
day -45	1.249	1.112
	(1.144)	(1.191)
day -44	1.280	1.219
	(1.152)	(1.232)
day -43	2.320**	2.162**
	(0.964)	(0.983)
day -42	2.001*	1.794
	(1.115)	(1.191)
day -41	2.518**	2.175*
	(1.139)	(1.104)
day -40	2.086**	1.871*
	(0.951)	(1.037)
day -39	1.640**	1.465*
	(0.725)	(0.853)
day -38	1.536**	1.423*
	(0.639)	(0.777)
day -37	1.560**	1.517**
	(0.587)	(0.687)
day -36	2.240***	2.185***
	(0.687)	(0.711)
day -35	2.386***	2.600***

	(0.806)	(0.865)
day -34	2.936***	3.012***
	(0.870)	(0.918)
day -33	2.951***	2.843***
	(0.868)	(0.811)
day -32	2.784***	2.658***
	(0.872)	(0.826)
day -31	2.213**	1.968**
	(0.830)	(0.785)
day -30	2.680***	2.296***
	(0.848)	(0.841)
day -29	2.462***	1.984**
	(0.825)	(0.843)
day -28	2.452***	1.944***
	(0.689)	(0.654)
day -27	2.351**	1.724**
	(0.930)	(0.834)
day -26	2.204**	1.605
	(1.024)	(0.961)
day -25	2.238**	1.672
	(1.085)	(1.062)
day -24	1.715	1.048
	(1.142)	(1.019)
day -23	1.756	1.225
	(1.844)	(1.740)
day -22	2.594	2.134
	(1.671)	(1.625)
day -21	2.802**	2.316*
	(1.383)	(1.369)
day -20	3.124**	2.720**
	(1.354)	(1.325)

day -19	2.607*	2.201
	(1.424)	(1.388)
day -18	2.795**	2.439**
	(1.256)	(1.193)
day -17	2.553**	2.308**
	(0.978)	(0.918)
day -16	2.348**	2.195**
	(0.988)	(0.938)
day -15	1.992***	1.767***
	(0.724)	(0.630)
day -14	2.116***	1.974***
	(0.757)	(0.639)
day -13	1.881**	1.669**
	(0.785)	(0.726)
day -12	1.586**	1.362**
	(0.686)	(0.640)
day -11	1.500*	1.207
	(0.869)	(0.757)
day -10	1.686**	1.299*
	(0.816)	(0.704)
day -9	2.419**	2.160**
	(1.132)	(0.978)
day -8	2.543**	2.272**
	(1.163)	(1.018)
day -7	1.940*	1.796*
	(1.060)	(0.942)
day -6	2.109*	1.948*
	(1.204)	(1.064)
day -5	2.424	2.247*
	(1.460)	(1.269)

day -4	2.376*	2.186**
	(1.253)	(1.058)
day -3	0.430	0.218
	(1.203)	(1.137)
day -2	0.723	0.507
	(1.155)	(1.133)
day -1	1.064	0.789
	(0.818)	(0.784)
day 1	1.323*	0.988
	(0.762)	(0.699)
day 2	1.728*	1.413
	(0.920)	(0.900)
day 3	1.763**	1.468*
	(0.808)	(0.773)
day 4	1.366	0.969
	(1.049)	(0.923)
day 5	1.135	0.779
	(1.068)	(0.965)
day 6	0.729	0.358
	(1.373)	(1.251)
day 7	0.718	0.371
	(1.460)	(1.363)
day 8	0.137	−0.238
	(1.131)	(1.042)
day 9	−0.354	−0.775
	(1.272)	(1.171)
day 10	0.035	−0.403
	(1.416)	(1.301)
day 11	−0.088	−0.536
	(1.154)	(1.042)
day 12	−0.052	−0.471

	(1.355)	(1.218)
day 13	0.482	0.009
	(1.096)	(0.899)
day 14	0.188	−0.275
	(0.920)	(0.774)
day 15	0.695	0.247
	(1.319)	(1.105)
day 16	0.044	−0.449
	(1.121)	(0.903)
day 17	−0.467	−0.982
	(1.109)	(0.898)
day 18	−0.718	−1.240
	(1.089)	(0.854)
day 19	−0.107	−0.535
	(1.344)	(1.119)
day 20	−0.628	−1.071
	(1.028)	(0.794)
day 21	−0.169	−0.658
	(1.244)	(1.005)
day 22	−0.039	−0.572
	(1.384)	(1.171)
day 23	−0.317	−0.825
	(1.248)	(1.052)
day 24	0.347	−0.198
	(1.519)	(1.331)
day 25	0.319	−0.262
	(1.193)	(1.010)
day 26	0.111	−0.315
	(0.988)	(0.874)
day 27	−0.455	−0.972
	(0.789)	(0.677)

day 28	-1.312^{*}	-1.930^{**}
	(0.726)	(0.734)
day 29	-0.766	-1.266
	(0.828)	(0.945)
day 30	-0.509	-0.997
	(0.576)	(0.619)
day 31	-1.454^{***}	-1.887^{***}
	(0.466)	(0.374)
day 32	-2.217^{***}	-2.688^{***}
	(0.757)	(0.683)
day 33	-1.526	-1.927^{**}
	(0.920)	(0.853)
day 34	-1.824^{***}	-2.235^{***}
	(0.653)	(0.475)
day 35	-2.050^{***}	-2.489^{***}
	(0.573)	(0.460)
day 36	-2.507^{***}	-2.970^{***}
	(0.875)	(0.787)
day 37	-2.228^{**}	-2.661^{***}
	(0.967)	(0.987)
day 38	-2.286^{*}	-2.760^{**}
	(1.233)	(1.237)
day 39	-2.673^{*}	-3.203^{**}
	(1.483)	(1.505)
day 40	-2.001	-2.401^{*}
	(1.416)	(1.358)
day 41	-2.209	-2.683^{*}
	(1.435)	(1.413)
day 42	-1.683	-2.173^{*}
	(1.183)	(1.129)

day 43	−1.577	−2.056*
	(1.264)	(1.217)
day 44	−1.509	−1.994**
	(0.984)	(0.929)
day 45	−1.907*	−2.382**
	(1.087)	(1.047)
Observations	10,398	10,398
R^2	0.945	0.943
Adjusted R^2	0.943	0.941
Residual Std. Error	1.637 (df = 10036)	1.670 (df = 10040)
Note:		*p<0.1; **p<0.05; ***p<0.01

S16 Table. Regression table. Regression statistics of mask adherence on confirmed new cases (1) and deaths (2)

	Dependent variable:	
	confirmed cases	deaths
	(1)	(2)
compliance	−1.675***	−0.017***
	(0.064)	(0.001)
new test rate	1,528.504***	14.615***
	(108.597)	(1.225)
precipitation	−0.419***	−0.003***
	(0.056)	(0.001)
temperature	−1.060***	−0.004***
	(0.058)	(0.001)
grocery and pharmacy	0.453***	0.002***
	(0.066)	(0.001)
parks	−0.170***	−0.002***
	(0.016)	(0.0002)
transit stations	−0.237***	−0.005***
	(0.043)	(0.0005)
workplaces	2.261***	0.015***
	(0.158)	(0.002)
residential	5.755***	0.021***
	(0.420)	(0.005)
grocery and pharmacy square	0.008***	0.0002***
	(0.002)	(0.00003)
retail and recreation square	0.0002	−0.00002
	(0.001)	(0.00002)
parks square	0.0004***	0.00000***
	(0.0001)	(0.00000)
transit stations square	−0.013***	−0.0002***
	(0.001)	(0.00001)
workplaces square	0.017***	0.0001***
	(0.003)	(0.00003)
residential square	−0.012	0.001***
	(0.025)	(0.0003)
Constant	191.442***	1.874***
	(5.384)	(0.061)
Observations	3,841	3,841
R^2	0.502	0.343
Note:	*p<0.1; **p<0.05; ***p<0.01	

S17 Table. Regression table. Multi-linear survey regression statistics of community mask adherence and community mask attitudes on COVID-19 Deaths and Cases in 69 countries over all waves.

	Dependent variable:			
	Cases	Deaths	Cases	Deaths
	(1)	(2)	(3)	(4)
Community Mask Attitudes	-0.605^{***}	-0.035^{***}		
	(0.110)	(0.003)		
Community Mask Adherence			-0.560^{***}	-0.036^{***}
			(0.121)	(0.003)
population density	-0.946^{***}	-0.023^{***}	-0.949^{***}	-0.023^{***}
	(0.021)	(0.001)	(0.021)	(0.001)
human development index	$5,157.998^{***}$	115.327^{***}	$5,169.927^{***}$	116.117^{***}
	(47.140)	(1.120)	(47.192)	(1.126)
new tests	34.811^{***}	0.867^{***}	34.662^{***}	0.854^{***}
	(1.709)	(0.031)	(1.710)	(0.031)
retail and recreation	-21.700^{***}	-0.233^{***}	-21.658^{***}	-0.230^{***}
	(0.380)	(0.007)	(0.379)	(0.007)
grocery and pharmacy	-1.225^{***}	-0.347^{***}	-1.289^{***}	-0.351^{***}
	(0.365)	(0.009)	(0.362)	(0.008)
parks	-0.097	-0.123^{***}	-0.041	-0.119^{***}
	(0.299)	(0.006)	(0.299)	(0.006)
transit stations	30.281^{***}	0.761^{***}	30.335^{***}	0.762^{***}
	(0.320)	(0.007)	(0.320)	(0.007)
workplaces	-2.403^{***}	-0.038^{***}	-2.482^{***}	-0.043^{***}
	(0.333)	(0.009)	(0.327)	(0.009)
residential	-5.306^{***}	0.217^{***}	-5.206^{***}	0.222^{***}
	(0.770)	(0.016)	(0.771)	(0.016)
Constant	$-2,844.272^{***}$	-54.236^{***}	$-2,855.701^{***}$	-54.736^{***}
	(39.495)	(0.935)	(39.213)	(0.919)
Observations	39,345	39,345	39,345	39,345
Note:				*p<0.1; **p<0.05; ***p<0.01

S18 Table. Regression table. Multi-linear survey regression statistics of community mask adherence on COVID-19 Cases in 69 countries per wave.

	Dependent variable: new cases																	
	(1)	(2)	(3)	(4)	(5)	(6)	(7)	(8)	(9)	(10)	(11)	(12)	(13)	(14)	(15)	(16)	(17)	(18)
Community Mask Adherence	−0.030**	−0.072***	−0.121***	−0.117***	−0.087***	−0.130***	−0.051***	−0.066**	0.010	−0.025	−0.061***	−0.084***	−0.321***	−0.172***	−0.101***	−0.032***	−0.022***	−0.056***
	(0.012)	(0.014)	(0.018)	(0.016)	(0.014)	(0.019)	(0.010)	(0.026)	(0.023)	(0.018)	(0.012)	(0.018)	(0.019)	(0.011)	(0.012)	(0.009)	(0.007)	(0.012)
population density	−0.050***	−0.050***	−0.052***	−0.079***	−0.072***	−0.072***	−0.046***	−0.018***	0.017***	−0.006**	−0.065***	−0.048***	−0.062***	−0.042***	−0.102***	−0.093***	−0.077***	−0.095***
	(0.002)	(0.003)	(0.003)	(0.004)	(0.003)	(0.005)	(0.002)	(0.002)	(0.004)	(0.003)	(0.004)	(0.003)	(0.004)	(0.002)	(0.002)	(0.002)	(0.002)	(0.002)
human development index	100.687***	115.536***	135.961***	135.703***	191.268***	256.050***	105.325***	135.468***	189.400***	306.656***	160.434***	159.018***	402.057***	312.388***	460.019***	446.776***	356.192***	516.993***
	(4.166)	(3.262)	(4.268)	(4.350)	(5.594)	(11.670)	(4.022)	(5.143)	(9.103)	(6.372)	(8.458)	(7.655)	(11.135)	(6.892)	(7.183)	(4.564)	(3.434)	(4.719)
new tests	65.363***	64.555***	50.779***	40.051***	16.954***	31.748***	43.813***	59.557***	79.160***	83.713***	88.774***	103.236***	83.721***	112.508***	64.205***	34.981***	21.839***	3.466***
	(0.630)	(0.351)	(0.457)	(0.443)	(0.645)	(0.719)	(0.466)	(0.840)	(0.688)	(0.370)	(0.752)	(0.450)	(0.618)	(0.439)	(0.374)	(0.222)	(0.193)	(0.171)
retail and recreation	0.452***	0.203***	0.052	−0.261***	−2.495***	−1.269***	−0.735***	−1.423***	−3.253***	−1.998***	2.290***	2.224***	1.582***	3.632***	2.200***	1.144***	−0.522***	−2.166***
	(0.031)	(0.024)	(0.032)	(0.052)	(0.058)	(0.094)	(0.048)	(0.141)	(0.137)	(0.076)	(0.093)	(0.069)	(0.061)	(0.045)	(0.041)	(0.034)	(0.026)	(0.038)
grocery and pharmacy	0.113***	0.160***	0.718***	1.185***	2.896***	0.620***	−0.184***	−0.440***	−1.090***	−1.593***	−1.979***	−0.723***	−1.097***	−2.587***	−2.259***	−1.613***	−0.942***	−0.129***
	(0.024)	(0.027)	(0.037)	(0.055)	(0.070)	(0.083)	(0.027)	(0.043)	(0.079)	(0.041)	(0.067)	(0.049)	(0.039)	(0.036)	(0.049)	(0.027)	(0.020)	(0.036)
parks	−0.425***	−0.580***	−0.425***	−0.418***	−0.177***	−0.492***	−0.846***	−0.814***	−0.657***	−1.641***	−1.812***	−1.931***	−0.565***	−0.663***	−0.772***	−1.373***	−0.279***	−0.004
	(0.011)	(0.008)	(0.008)	(0.013)	(0.018)	(0.019)	(0.015)	(0.031)	(0.061)	(0.040)	(0.050)	(0.046)	(0.038)	(0.040)	(0.034)	(0.031)	(0.012)	(0.030)
transit stations	0.091**	−0.019	0.331***	0.070**	0.403***	0.672***	0.774***	0.740***	1.533***	2.228***	1.053***	0.883***	0.809***	1.067***	2.782***	3.151***	2.565***	3.034***
	(0.036)	(0.029)	(0.027)	(0.034)	(0.036)	(0.078)	(0.044)	(0.062)	(0.083)	(0.060)	(0.087)	(0.064)	(0.058)	(0.042)	(0.042)	(0.032)	(0.024)	(0.032)
workplaces	1.762***	0.812***	1.291***	1.263***	−0.243***	2.137***	0.872***	0.747***	−1.109***	0.200***	1.333***	0.091	−0.044	−0.180***	0.309***	0.219***	−0.573***	−0.248***
	(0.056)	(0.047)	(0.052)	(0.046)	(0.050)	(0.060)	(0.054)	(0.070)	(0.083)	(0.051)	(0.053)	(0.080)	(0.051)	(0.051)	(0.052)	(0.034)	(0.033)	(0.033)
residential	4.831***	2.201***	5.527***	6.105***	2.589***	4.890***	1.449***	−0.607**	−4.416***	−0.615***	4.830***	1.147***	1.799***	2.119***	2.254***	2.243***	−0.294***	−0.521***
	(0.172)	(0.134)	(0.168)	(0.141)	(0.172)	(0.222)	(0.068)	(0.248)	(0.299)	(0.179)	(0.209)	(0.178)	(0.235)	(0.098)	(0.100)	(0.098)	(0.061)	(0.077)
Constant	−54.797***	−50.147***	−68.533***	−82.171***	−140.882***	−149.645***	−49.379***	−82.623***	−153.557***	−199.698***	−55.937***	−70.225***	−212.585***	−130.682***	−184.646***	−203.152***	−174.432***	−285.570***
	(2.897)	(2.317)	(3.403)	(3.166)	(4.177)	(8.739)	(3.045)	(4.165)	(7.241)	(4.654)	(5.009)	(5.517)	(7.313)	(4.298)	(4.767)	(3.233)	(2.479)	(3.921)
Observations	27,387	33,991	30,288	24,544	22,511	8,663	19,985	18,960	18,292	36,726	41,825	38,405	42,088	39,348	38,576	39,478	39,127	39,345
Note:																		*p<0.1; **p<0.05; ***p<0.01

S19 Table. Regression table. Multi-linear survey regression statistics of community mask attitudes on COVID-19 Cases in 69 countries per wave.

	Dependent variable: new cases																	
	(1)	(2)	(3)	(4)	(5)	(6)	(7)	(8)	(9)	(10)	(11)	(12)	(13)	(14)	(15)	(16)	(17)	(18)
Community Mask Attitudes	−0.052***	−0.067***	−0.115***	−0.090***	−0.086***	−0.101***	−0.055***	−0.053***	−0.025	−0.116***	−0.085***	−0.140***	−0.329***	−0.156***	−0.085***	−0.051***	−0.034***	−0.061***
	(0.014)	(0.014)	(0.015)	(0.014)	(0.013)	(0.014)	(0.007)	(0.013)	(0.016)	(0.017)	(0.014)	(0.018)	(0.021)	(0.011)	(0.011)	(0.009)	(0.007)	(0.011)
population density	−0.045***	−0.052***	−0.051***	−0.076***	−0.073***	−0.070***	−0.043***	−0.018***	0.022***	−0.006**	−0.065***	−0.047***	−0.062***	−0.041***	−0.102***	−0.093***	−0.077***	−0.095***
	(0.002)	(0.003)	(0.003)	(0.004)	(0.003)	(0.003)	(0.001)	(0.001)	(0.002)	(0.003)	(0.004)	(0.003)	(0.004)	(0.002)	(0.002)	(0.002)	(0.002)	(0.002)
human development index	100.088***	112.937***	135.549***	128.050***	196.577***	261.561***	107.297***	129.944***	177.052***	310.199***	159.394***	158.539***	394.153***	306.217***	456.664***	445.970***	355.807***	515.800***
	(4.225)	(3.337)	(4.223)	(4.616)	(5.636)	(7.863)	(2.572)	(3.192)	(5.768)	(6.210)	(8.392)	(7.461)	(10.992)	(6.905)	(7.160)	(4.560)	(3.411)	(4.714)
new tests	65.956***	64.334***	50.763***	39.308***	15.361***	31.107***	43.780***	60.056***	79.668***	83.350***	88.672***	103.014***	83.655***	112.574***	64.257***	34.970***	21.832***	3.481***
	(0.627)	(0.378)	(0.441)	(0.492)	(0.672)	(0.491)	(0.300)	(0.475)	(0.450)	(0.370)	(0.758)	(0.447)	(0.616)	(0.439)	(0.373)	(0.221)	(0.193)	(0.171)
retail and recreation	0.438***	0.200***	0.064*	−0.294***	−2.542***	−1.216***	−0.728***	−1.180***	−3.245***	−1.997***	2.281***	2.199***	1.566***	3.618***	2.196***	1.144***	−0.525***	−2.170***
	(0.031)	(0.026)	(0.033)	(0.054)	(0.059)	(0.064)	(0.031)	(0.086)	(0.091)	(0.076)	(0.093)	(0.069)	(0.061)	(0.045)	(0.041)	(0.034)	(0.026)	(0.038)
grocery and pharmacy	0.096***	0.123***	0.679***	1.225***	2.979***	0.625***	−0.181***	−0.498***	−1.121***	−1.571***	−1.972***	−0.710***	−1.092***	−2.587***	−2.263***	−1.604***	−0.935***	−0.123***
	(0.024)	(0.028)	(0.037)	(0.062)	(0.069)	(0.056)	(0.018)	(0.027)	(0.051)	(0.041)	(0.067)	(0.048)	(0.039)	(0.036)	(0.048)	(0.027)	(0.020)	(0.036)
parks	−0.446***	−0.575***	−0.441***	−0.413***	−0.153***	−0.521***	−0.848***	−0.860***	−0.649***	−1.639***	−1.806***	−1.927***	−0.574***	−0.681***	−0.783***	−1.376***	−0.281***	−0.010
	(0.011)	(0.008)	(0.009)	(0.013)	(0.017)	(0.012)	(0.010)	(0.020)	(0.040)	(0.040)	(0.049)	(0.046)	(0.038)	(0.040)	(0.033)	(0.031)	(0.012)	(0.030)
transit stations	0.083**	−0.027	0.387***	0.023	0.397***	0.727***	0.781***	0.636***	1.390***	2.195***	1.041***	0.874***	0.794***	1.071***	2.788***	3.137***	2.558***	3.028***
	(0.036)	(0.033)	(0.027)	(0.034)	(0.036)	(0.047)	(0.028)	(0.039)	(0.053)	(0.059)	(0.086)	(0.063)	(0.058)	(0.042)	(0.042)	(0.032)	(0.024)	(0.032)
workplaces	1.704***	0.890***	1.223***	1.172***	−0.183***	2.051***	0.859***	0.769***	−1.131***	0.187***	1.340***	0.087	−0.031	−0.176***	0.308***	0.218***	−0.571***	−0.240***
	(0.056)	(0.050)	(0.051)	(0.049)	(0.051)	(0.038)	(0.036)	(0.048)	(0.053)	(0.052)	(0.054)	(0.080)	(0.051)	(0.051)	(0.052)	(0.034)	(0.033)	(0.033)
residential	4.583***	2.270***	5.474***	5.858***	2.769***	4.875***	1.463***	−0.543***	−4.819***	−0.639***	4.831***	1.140***	1.743***	2.079***	2.226***	2.232***	−0.296***	−0.531***
	(0.170)	(0.139)	(0.166)	(0.148)	(0.176)	(0.140)	(0.047)	(0.157)	(0.207)	(0.180)	(0.207)	(0.175)	(0.232)	(0.098)	(0.099)	(0.097)	(0.061)	(0.077)
Constant	−53.376***	−46.485***	−67.929***	−79.256***	−144.499***	−155.089***	−51.282***	−77.832***	−143.545***	−196.108***	−53.360***	−65.904***	−205.146***	−127.094***	−183.222***	−201.366***	−173.455***	−284.427***
	(2.971)	(2.502)	(3.431)	(3.349)	(4.324)	(5.967)	(2.000)	(2.646)	(4.736)	(4.662)	(4.862)	(5.490)	(7.331)	(4.335)	(4.801)	(3.282)	(2.497)	(3.949)
Observations	26,707	32,941	29,493	23,816	22,007	17,195	40,071	37,862	36,959	36,774	41,825	38,405	42,088	39,348	38,576	39,478	39,127	39,345
Note:																		*p<0.1; **p<0.05; ***p<0.01

S20 Table. Regression table. Multi-linear survey regression statistics of community mask adherence on COVID-19 deaths in 69 countries per wave.

	Dependent variable: new deaths																	
	(1)	(2)	(3)	(4)	(5)	(6)	(7)	(8)	(9)	(10)	(11)	(12)	(13)	(14)	(15)	(16)	(17)	(18)
Community Mask Adherence	−0.004***	−0.005***	−0.006***	−0.005***	−0.004***	−0.005***	−0.005***	−0.004***	−0.004***	−0.002***	−0.003***	−0.004***	−0.007***	−0.005***	−0.007***	−0.006***	−0.004***	−0.004***
	(0.0004)	(0.0004)	(0.001)	(0.0005)	(0.0004)	(0.001)	(0.001)	(0.0004)	(0.0004)	(0.001)	(0.0004)	(0.001)	(0.0004)	(0.0004)	(0.001)	(0.0005)	(0.0003)	(0.0003)
population density	−0.002***	−0.002***	−0.002***	−0.003***	−0.002***	−0.002***	−0.002***	−0.001***	−0.001***	−0.001***	−0.0004***	−0.001***	−0.0005***	−0.001***	−0.001***	−0.002***	−0.002***	−0.002***
	(0.0001)	(0.0001)	(0.0001)	(0.0001)	(0.0001)	(0.0002)	(0.0001)	(0.0001)	(0.0001)	(0.0001)	(0.0001)	(0.0001)	(0.0001)	(0.0001)	(0.0001)	(0.0001)	(0.0001)	(0.0001)
human development index	4.711***	5.640***	6.052***	6.045***	5.567***	7.828***	5.938***	5.664***	4.857***	9.108***	5.218***	11.921***	13.507***	14.706***	8.695***	11.238***	10.280***	11.612***
	(0.139)	(0.124)	(0.140)	(0.153)	(0.159)	(0.381)	(0.188)	(0.144)	(0.171)	(0.162)	(0.183)	(0.218)	(0.201)	(0.199)	(0.193)	(0.162)	(0.117)	(0.113)
new tests	0.257***	0.599***	0.482***	0.422***	−0.169***	0.191***	0.184***	0.311***	0.444***	0.448***	0.936***	0.837***	0.679***	0.864***	1.233***	0.933***	0.479***	0.085***
	(0.023)	(0.014)	(0.016)	(0.016)	(0.016)	(0.027)	(0.015)	(0.010)	(0.010)	(0.010)	(0.012)	(0.011)	(0.009)	(0.009)	(0.009)	(0.008)	(0.007)	(0.003)
retail and recreation	−0.006***	0.00004	−0.009***	−0.004**	−0.046***	−0.014***	−0.039***	−0.040***	−0.021***	−0.128***	−0.018***	−0.001	−0.047***	−0.079***	−0.083***	−0.029***	−0.013***	−0.023***
	(0.001)	(0.001)	(0.001)	(0.002)	(0.001)	(0.003)	(0.002)	(0.002)	(0.002)	(0.002)	(0.002)	(0.002)	(0.001)	(0.001)	(0.002)	(0.001)	(0.001)	(0.001)
grocery and pharmacy	−0.008***	−0.008***	0.0003	0.013***	0.038***	0.011***	−0.004***	−0.002*	−0.018***	−0.007***	−0.026***	0.022***	0.017***	−0.027***	−0.041***	−0.065***	−0.044***	−0.035***
	(0.001)	(0.001)	(0.001)	(0.002)	(0.002)	(0.002)	(0.001)	(0.001)	(0.001)	(0.001)	(0.002)	(0.001)	(0.001)	(0.001)	(0.002)	(0.001)	(0.001)	(0.001)
parks	−0.008***	−0.015***	−0.012***	−0.017***	−0.006***	−0.017***	−0.020***	−0.022***	−0.020***	−0.046***	−0.047***	−0.038***	−0.015***	−0.007***	−0.006***	−0.022***	−0.009***	−0.012***
	(0.0003)	(0.0003)	(0.0003)	(0.0004)	(0.0004)	(0.001)	(0.001)	(0.001)	(0.001)	(0.001)	(0.001)	(0.001)	(0.001)	(0.002)	(0.002)	(0.001)	(0.001)	(0.001)
transit stations	0.009***	0.009***	0.014***	0.007***	0.007***	0.018***	0.019***	0.023***	0.012***	0.072***	−0.012***	0.026***	0.048***	0.102***	0.074***	0.077***	0.060***	0.076***
	(0.001)	(0.001)	(0.001)	(0.001)	(0.001)	(0.002)	(0.001)	(0.001)	(0.001)	(0.001)	(0.002)	(0.001)	(0.001)	(0.001)	(0.002)	(0.001)	(0.001)	(0.001)
workplaces	0.026***	0.014***	0.016***	0.014***	−0.016***	0.016***	0.028***	0.016***	0.011***	0.013***	0.040***	−0.023***	−0.044***	−0.060***	−0.035***	0.013***	−0.008***	−0.004***
	(0.002)	(0.001)	(0.001)	(0.001)	(0.001)	(0.002)	(0.002)	(0.002)	(0.002)	(0.001)	(0.002)	(0.002)	(0.001)	(0.002)	(0.003)	(0.002)	(0.001)	(0.001)
residential	0.094***	0.072***	0.105***	0.127***	0.045***	0.098***	0.049***	0.006	0.036***	−0.042***	0.039***	0.002	−0.078***	−0.095***	−0.108***	0.024***	0.015***	0.022***
	(0.005)	(0.004)	(0.004)	(0.004)	(0.004)	(0.006)	(0.004)	(0.004)	(0.004)	(0.004)	(0.005)	(0.004)	(0.004)	(0.003)	(0.003)	(0.002)	(0.002)	(0.002)
Constant	−2.301***	−2.609***	−2.915***	−3.392***	−3.445***	−4.322***	−3.032***	−3.179***	−2.630***	−6.084***	−3.134***	−7.612***	−8.511***	−8.312***	−4.262***	−5.020***	−4.620***	−5.474***
	(0.090)	(0.079)	(0.097)	(0.107)	(0.117)	(0.269)	(0.144)	(0.108)	(0.127)	(0.119)	(0.135)	(0.148)	(0.139)	(0.133)	(0.148)	(0.131)	(0.091)	(0.092)
Observations	27,387	33,991	30,288	24,544	22,511	8,663	19,985	18,960	18,292	36,726	41,825	38,405	42,088	39,348	38,576	39,478	39,127	39,345
Note:																		*p<0.1; **p<0.05; ***p<0.01

S21 Table. Regression table. Multi-linear survey regression statistics of community mask attitudes on COVID-19 deaths in 69 countries per wave.

	Dependent variable: new deaths																	
	(1)	(2)	(3)	(4)	(5)	(6)	(7)	(8)	(9)	(10)	(11)	(12)	(13)	(14)	(15)	(16)	(17)	(18)
Community Mask Attitudes	−0.004***	−0.004***	−0.005***	−0.004***	−0.003***	−0.004***	−0.004***	−0.003***	−0.003***	−0.003***	−0.004***	−0.006***	−0.007***	−0.005***	−0.005***	−0.005***	−0.004***	−0.003***
	(0.001)	(0.0004)	(0.0005)	(0.0005)	(0.0004)	(0.0005)	(0.0003)	(0.0003)	(0.0003)	(0.0004)	(0.0005)	(0.001)	(0.0004)	(0.0004)	(0.0005)	(0.0004)	(0.0004)	(0.0003)
population density	−0.002***	−0.002***	−0.002***	−0.002***	−0.002***	−0.002***	−0.002***	−0.001***	−0.001***	−0.001***	−0.0004***	−0.001***	−0.0004***	−0.001***	−0.001***	−0.002***	−0.002***	−0.002***
	(0.0001)	(0.0001)	(0.0001)	(0.0001)	(0.0001)	(0.0001)	(0.0001)	(0.00004)	(0.00005)	(0.0001)	(0.0001)	(0.0001)	(0.0001)	(0.0001)	(0.0001)	(0.0001)	(0.0001)	(0.0001)
human development index	4.734***	5.483***	5.855***	5.761***	5.614***	7.902***	5.942***	5.407***	4.552***	9.082***	5.159***	11.863***	13.340***	14.529***	8.466***	11.073***	10.172***	11.533***
	(0.141)	(0.124)	(0.137)	(0.168)	(0.155)	(0.246)	(0.122)	(0.089)	(0.109)	(0.158)	(0.182)	(0.208)	(0.198)	(0.201)	(0.192)	(0.160)	(0.117)	(0.112)
new tests	0.253***	0.592***	0.503***	0.396***	−0.215***	0.166***	0.183***	0.321***	0.449***	0.444***	0.932***	0.830***	0.678***	0.866***	1.237***	0.935***	0.481***	0.087***
	(0.024)	(0.014)	(0.015)	(0.018)	(0.016)	(0.016)	(0.010)	(0.007)	(0.007)	(0.009)	(0.012)	(0.011)	(0.009)	(0.009)	(0.009)	(0.008)	(0.007)	(0.003)
retail and recreation	−0.008***	0.001	−0.008***	−0.005***	−0.048***	−0.015***	−0.037***	−0.039***	−0.021***	−0.128***	−0.018***	−0.002	−0.048***	−0.080***	−0.084***	−0.029***	−0.014***	−0.023***
	(0.001)	(0.001)	(0.001)	(0.002)	(0.001)	(0.002)	(0.001)	(0.001)	(0.001)	(0.002)	(0.002)	(0.002)	(0.001)	(0.001)	(0.002)	(0.001)	(0.001)	(0.001)
grocery and pharmacy	−0.010***	−0.008***	−0.0001	0.015***	0.040***	0.009***	−0.005***	−0.002***	−0.019***	−0.006***	−0.026***	0.022***	0.017***	−0.027***	−0.042***	−0.064***	−0.043***	−0.035***
	(0.001)	(0.001)	(0.001)	(0.002)	(0.002)	(0.002)	(0.001)	(0.001)	(0.001)	(0.001)	(0.002)	(0.001)	(0.001)	(0.001)	(0.002)	(0.001)	(0.001)	(0.001)
parks	−0.009***	−0.014***	−0.013***	−0.016***	−0.005***	−0.018***	−0.020***	−0.022***	−0.020***	−0.046***	−0.047***	−0.038***	−0.015***	−0.008***	−0.006***	−0.022***	−0.009***	−0.012***
	(0.0003)	(0.0003)	(0.0003)	(0.0004)	(0.0004)	(0.0004)	(0.0004)	(0.0004)	(0.001)	(0.001)	(0.001)	(0.001)	(0.001)	(0.002)	(0.002)	(0.001)	(0.001)	(0.001)
transit stations	0.009***	0.010***	0.015***	0.005***	0.008***	0.020***	0.019***	0.021***	0.010***	0.072***	−0.013***	0.026***	0.048***	0.102***	0.075***	0.077***	0.060***	0.076***
	(0.001)	(0.001)	(0.001)	(0.001)	(0.001)	(0.001)	(0.001)	(0.001)	(0.001)	(0.001)	(0.002)	(0.001)	(0.001)	(0.001)	(0.002)	(0.001)	(0.001)	(0.001)
workplaces	0.023***	0.016***	0.016***	0.013***	−0.016***	0.014***	0.028***	0.017***	0.013***	0.013***	0.040***	−0.024***	−0.044***	−0.060***	−0.035***	0.013***	−0.008***	−0.004***
	(0.002)	(0.001)	(0.001)	(0.001)	(0.001)	(0.001)	(0.001)	(0.001)	(0.001)	(0.001)	(0.002)	(0.002)	(0.001)	(0.002)	(0.003)	(0.002)	(0.001)	(0.001)
residential	0.086***	0.079***	0.099***	0.121***	0.049***	0.097***	0.052***	0.005*	0.036***	−0.042***	0.039***	0.002	−0.080***	−0.096***	−0.110***	0.022***	0.015***	0.022***
	(0.005)	(0.004)	(0.004)	(0.004)	(0.004)	(0.004)	(0.003)	(0.003)	(0.003)	(0.004)	(0.005)	(0.004)	(0.004)	(0.003)	(0.003)	(0.002)	(0.002)	(0.002)
Constant	−2.317***	−2.520***	−2.804***	−3.281***	−3.517***	−4.409***	−3.071***	−3.019***	−2.466***	−6.018***	−3.017***	−7.458***	−8.364***	−8.200***	−4.203***	−4.921***	−4.552***	−5.424***
	(0.093)	(0.083)	(0.095)	(0.117)	(0.117)	(0.177)	(0.094)	(0.069)	(0.084)	(0.119)	(0.136)	(0.146)	(0.140)	(0.137)	(0.150)	(0.130)	(0.092)	(0.093)
Observations	26,707	32,941	29,493	23,816	22,007	17,195	40,071	37,862	36,959	36,774	41,825	38,405	42,088	39,348	38,576	39,478	39,127	39,345

Note: *p<0.1; **p<0.05; ***p<0.01
